# TDP-43 induces p53-mediated cell death of cortical progenitors and immature neurons

**DOI:** 10.1038/s41598-018-26397-2

**Published:** 2018-05-25

**Authors:** Miriam A. Vogt, Zahra Ehsaei, Philip Knuckles, Adrian Higginbottom, Michaela S. Helmbrecht, Tilo Kunath, Kevin Eggan, Luis A. Williams, Pamela J. Shaw, Wolfgang Wurst, Thomas Floss, Andrea B. Huber, Verdon Taylor

**Affiliations:** 1 Department of Biomedicine, University of Basel, Mattenstrasse 28, 4058 Basel, Switzerland; 2 Friedrich Miescher Institute for Biomedical Research, Maulbeerstrasse 66, 4058 Basel, Switzerland; 3 Sheffield Institute for Translational Neuroscience (SITraN), University of Sheffield, 385A Glossop Road, Sheffield, S10 2HQ UK; 4 Helmholtz Zentrum München, Ingolstädter Landstrasse 1, 85764 Neuherberg, Germany; 5 MRC Centre for Regenerative Medicine, The University of Edinburgh, 5 Little France Drive, Edinburgh, EH16 4UU UK; 6 Harvard Stem Cell Institute, Harvard University, Howard Hughes Medical Institute, 7 Divinity Avenue, Cambridge, MA 02138 USA; 7 Present Address: Ludwig-Maximilians University Munich, Feodor-Lynen-Strasse 17, 81377 München, Germany; 8 Present Address: ETH Zurich, Department of Biosystems Science and Engineering, Mattenstrasse 26, 4058 Basel, Switzerland

## Abstract

TAR DNA-binding protein 43 (TDP-43) is a key player in neurodegenerative diseases including frontotemporal lobar degeneration (FTLD) and amyotrophic lateral sclerosis (ALS). Accumulation of TDP-43 is associated with neuronal death in the brain. How increased and disease-causing mutant forms of TDP-43 induce cell death remains unclear. Here we addressed the role of TDP-43 during neural development and show that reduced TDP-43 causes defects in neural stem/progenitor cell proliferation but not cell death. However, overexpression of wild type and TDP-43^A315T^ proteins induce p53-dependent apoptosis of neural stem/progenitors and human induced pluripotent cell (iPS)-derived immature cortical neurons. We show that TDP-43 induces expression of the proapoptotic BH3-only genes *Bbc3* and *Bax*, and that p53 inhibition rescues TDP-43 induced cell death of embryonic mouse, and human cortical neurons, including those derived from TDP-43^G298S^ ALS patient iPS cells. Hence, an increase in wild type and mutant TDP-43 induces p53-dependent cell death in neural progenitors developing neurons and this can be rescued. These findings may have important implications for accumulated or mutant TDP-43 induced neurodegenerative diseases.

## Introduction

The nucleotide binding protein TDP-43 regulates a multitude of cellular processes including gene transcription, RNA splicing, mRNA stability, localization and translation, and microRNA biogenesis^1^. TDP-43 is associated with most cases of FTLD and ALS. Point mutations and elevated TDP-43 levels cause FTLD-TDP coupled with neuronal death. Affected neurons have nuclear depletion and cytoplasmic accumulation of TDP-43. Interestingly, accumulation of TDP-43 in neurons is characteristic of many neurodegenerative diseases in addition to FTLD-TDP and ALS, including Alzheimer’s disease. TDP-43 seems to play roles in the onset and progression of neurodegeneration but mechanistically how it triggers and contributes to disease and cell death is not known.

TDP-43 was originally identified as a factor binding to the TAR DNA of human immunodeficiency virus where it is implicated in transcriptional regulation^2^. It belongs to the family of heterogeneous nuclear ribonucleoproteins (hnRNP) and is ubiquitously expressed. Deletion of the *Tardbp* (the gene encoding TDP-43) in mice leads to early embryonic lethality between E3.5 and E6.5^3,4^ indicating an important role during early development. TDP-43 contains two RNA-recognition motifs (RRMs), RRM-1 and RRM-2, and a glycine-rich region at its C-terminus^5^. The RRM-1 is necessary and sufficient for nucleic acid binding to single stranded RNA at GU-rich sequences^6^. The C-terminus of TDP-43 is necessary for the formation of hnRNP-rich complexes^7^ and contains most of the TDP-43 point mutations identified in FTLD and ALS patients. TDP-43 is localized predominantly nuclear in cells and has both a nuclear localization sequence (NLS) and a predicted nuclear export sequence (NES) and seems to be continuously shuttled between the two cellular compartments^8^.

TDP-43 is one of the main components of cytoplasmic inclusions, which are a characteristic feature of many neurodegenerative disorders. Apoptotic neurons that display cytoplasmic inclusions show a partial loss of TDP-43 in the nucleus^9^, which was suggested to drive, at least in part, disease pathogenesis. However, the cause and function of TDP-43 aggregates remains to be shown unequivocally. In mice, robust cytoplasmic TDP-43 aggregation is associated with dramatic neuron loss and features of human pathology, which can be reversed by increased clearance of TDP-43^10^. Interestingly, mislocalization of TDP-43 to the cytoplasm of mouse neurons is sufficient to induce apoptosis even in the absence of aggregation, suggesting that cytoplasmic TDP-43 aggregates may not be necessary to induce cell death and early mortality in mice^9,11–13^.

Aberrant TDP-43 causes pleiotropic effects in cells and results in extensive changes in splicing and RNA metabolism^14^. Cross-linked immunoprecipitation and RNA sequencing (CLIP-Seq) revealed that TDP-43 can bind thousands of RNAs via a UG-rich consensus sequence in the 3′ untranslated regions of target RNAs^15–17^. Whereas the RNAs bound by TDP43 in the mouse brain are relatively consistent in the different analyses, TDP-43 targets vary considerably between cell types^16,17^.

Aggregates in diseased neurons contain hyper-phosphorylated and fragmented TDP-43 protein. Interestingly, TDP-43 can be cleaved by caspases^18^, and other factors of the apoptosis pathway including Bim, Bax and Bcl may be involved in TDP-43-induced cell death^19^. Components of the proapoptotic pathway are downstream targets of p53 and elevated p53 levels have been detected in affected neurons of ALS patients^20,21^. However, the absence of p53 in a transgenic mouse model for ALS (hSOD1^G93A^) did not rescue apoptosis, suggesting that cell death in these animals occurred in a p53-independent manner^22,23^. Although aberrant TDP-43 expression is associated with stress responses^24^, a causal link between p53 and TDP-43 induced cell death has not been reported. TDP-43 is expressed in the developing and adult brain, therefore, we addressed the role of TDP-43 during development of the telencephalon by gain- and loss-of-function experiments. We thereby hoped to gain insights into TDP-43 functions in the formation and maintenance of the nervous system.

Here we show that expression of TDP-43 and its mutant form TDP-43^A315T^ results in p53-mediated apoptosis in neural stem/progenitor cells and immature neurons of the developing mouse telencephalon. In addition, we observed cell death of cortical neurons derived from human iPS cells following TDP-43 expression and found that this neuronal death could also be rescued by p53-inhibition. Expression of the proapoptotic BH3-only genes *Bbc3* and *Bax* was increased in mice and human neural cells as a result of aberrant TDP-43 expression, supporting a role for p53 in the TDP-43 induced cell death. Furthermore, we show that TDP-43 is associated with the mRNA of *Cdkn1a* and increases Cdkn1a levels, likely explaining the altered neural stem/progenitor cell cycle regulation following TDP-43 and TDP-43^A315T^ expression.

## Results

### TDP-43 controls cell cycle, neurogenesis and is toxic for neural progenitors

**Figure 1:**
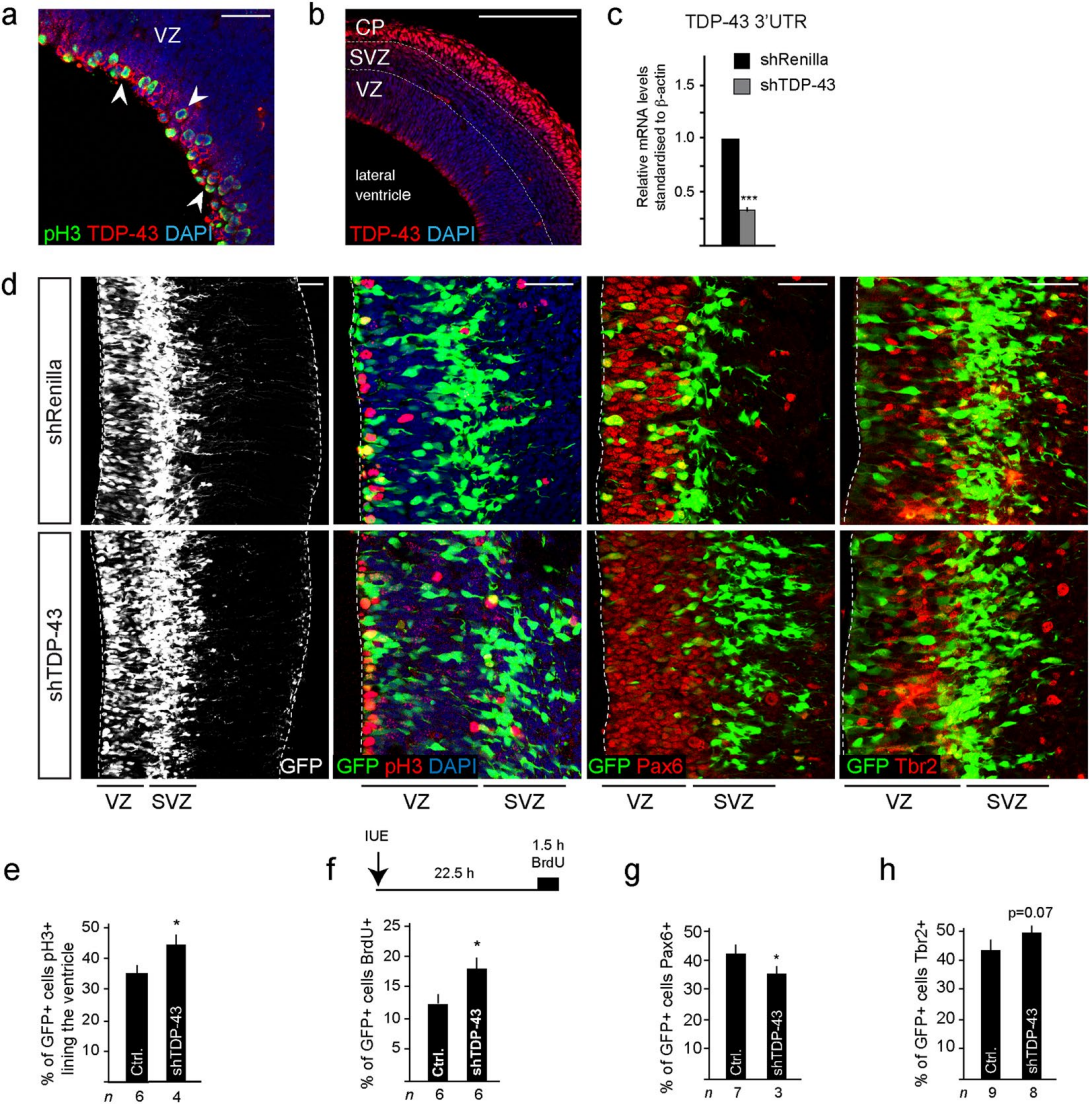
TDP-43 knockdown (KD) decreases Pax6^+^ neural stem/progenitors and affects cell cycle. (**a**) TDP-43 is expressed by ventricular zone (VZ) progenitors including by cells in mitosis (arrows). (**b**) TDP-43 is highly expressed by neurons in the cortical plate (CP) at e14.5. (**c**) TDP-43 KD (shTDP-43) by *in utero* electroporation (IUE) of neural stem/progenitors at e13.5 and analyzed at e14.5 reduces TDP-43 mRNA levels by 70% (^***^P < 0.001) compared to control (shRenilla). (**d**) TDP-43 KD (GFP^+^) cells survive in the VZ and differentiate to generate Tbr2^+^ basal progenitors in the subventricular zone (SVZ) similar to control transfected cells (shRenilla). (**e**) The proportion of cells lining the ventricle that are pH3^+^ increases following TDP-43 KD (shTDP-43) compared to control cells (shRenilla: Ctrl.), suggesting accumulation in M-phase of cell cycle. Values are shown as the proportion of transfected cells (GFP^+^). (**f**) Scheme of the BrdU labeling procedure in e13.5 mice with a BrdU pulse 22.5 hours post IUE and 1.5 hours prior to killing. The proportion of GFP^+^ cells in the VZ that are BrdU labeled following TDP-43 KD (shTDP-43) increases compared to control cells (shRenilla: Ctrl.), suggesting increased S-phase entry. Values are shown as the proportion of transfected cells (GFP^+^). (**g**) The proportion of GFP^+^ cells that are Pax6^+^ following TDP-43 KD (shTDP-43) reduces compared to control cells (shRenilla: Ctrl.). Values are shown as the proportion of transfected cells (GFP^+^). (**h**) The proportion of GFP^+^ cells that are Tbr2^+^ following TDP-43 KD (shTDP-43) is unaffected compared to control cells (shRenilla: Ctrl.). Values are shown as the proportion of transfected cells (GFP^+^). Scale bars in a and d = 25 µm, in b = 100 µm. Dashed line marks the telencephalic vesicle lining. tTest ^*^P < 0.05, ^***^< 0.001. ventricular zone (VZ), subventricular zone (SVZ), cortical plate (CP).

*Tardbp* is expressed by neural progenitors in the developing central nervous system (Supplementary Fig. 1a)^3^. In the developing telencephalon at e14.5, TDP-43 protein is prominent in ventricular zone stem/progenitor cells including by those in M-phase of the cell cycle at the ventricular surface where expression partially overlaps with phospho-Histone H3 (pH3) (Fig. 1a, arrowheads, Supplementary Fig. 1b). TDP-43 protein expression is also prominent in differentiating neurons in the cortical plate (Fig. 1b).

To address the functions of TDP-43 during forebrain development, we knocked-down (KD) TDP-43 *in vivo* targeting the ventricular zone stem and progenitor cells by *in utero* electroporation (IUE) with hairpin RNAs targeting its mRNA (shTDP-43). TDP-43 mRNA and protein levels were reduced in neural progenitors by up to ~70% 48 hours after transfection (Fig. 1c, Supplementary Fig. 2a–d**)**. The percentage of transfected cells that expressed detectable levels of TDP-43 *in vivo* reduced from 93.9 +/− 4.2% in controls to 3.7 +/− 2.5% in shTDP-43 transfected embryos (Supplementary Fig. 2c,d). TDP-43 KD increased the number of progenitors in M-phase within 24 hours (pH3^+^: Fig. 1d,e), as well as those entering S-phase (BrdU labeled) (Fig. 1f). These effects were consistent with the expression of TDP-43 by mitotic cells in the developing telencephalon. TDP-43 KD also reduced the number of Pax6^+^ ventricular zone progenitors and increased the generation of Tbr2^+^ basal progenitors slightly (p = 0.07) (Fig. 1d,g,h and Supplementary Fig. 2e–h). After 48 hours, the increase in BrdU^+^ cells in the ventricular zone following TDP-43 KD was still evident but the formation of Tbr2^+^ basal progenitors had returned to normal and there were no obvious effects on cell survival (Supplementary Fig. 2i,j and data not shown).

**Figure 2:**
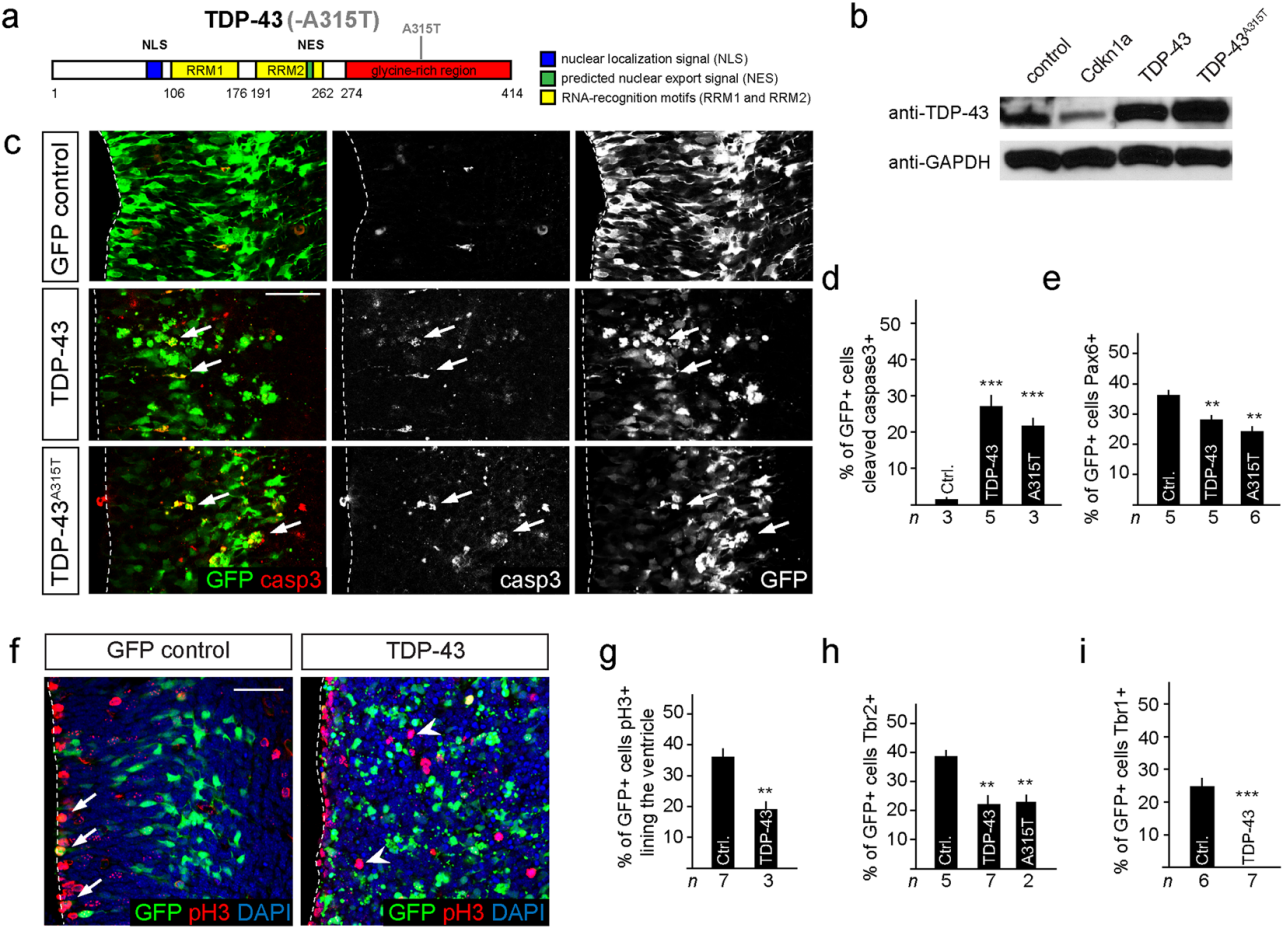
Expression of TDP-43 and TDP-43^A315T^ induce p53-dependent apoptosis of neural progenitors. (**a**) Scheme of human TDP-43 and TDP-43^A315T^ with a single point mutation in the glycine-rich C-terminal region, nuclear localization signal (NLS), nuclear export signal (NES), RNA recognition motif (RRM). (**b**) Transfection of neural progenitors results in a 2.4-fold expression of TDP-43 and 2.8-fold for TDP-43^A315T^ compared to endogenous levels. Expression of Cdkn1 reduces endogenous TDP-43 levels suggesting a reciprocal interaction with TDP-43. (**c**) Expression of TDP-43 and TDP-43^A315T^ activates caspase-3 (arrows) and drives cells in the ventricular zone into apoptosis within 24 hours. (**d**) TDP-43 and TDP-43^A315T^ expression induce caspase-3 activity and apoptosis in transfected cells (GFP^+^). Values are shown as the proportion of transfected cells (GFP^+^). (**e**) TDP-43 and TDP-43^A315T^ expression reduce the proportion of Pax6^+^GFP^+^ progenitors compared to control transfected cells (Ctrl.). Values are shown as the proportion of transfected cells (GFP^+^). (**f**,**g**) TDP-43 expression causes mislocalization of GFP^+^pH3^+^ cells away from the ventricular lining (GFP control: arrows) to more basal locations (TDP-43: arrowheads) and reduces the proportion of ventricular progenitors in M-phase (pH3^+^) compared to control transfected cells (Ctrl.). Values are shown as the proportion of transfected cells (GFP^+^). (**h**) TDP-43 and TDP-43^A315T^ expression reduce differentiation to Tbr2^+^ basal progenitors compared to control transfected cells (Ctrl.). Values are shown as the proportion of transfected cells (GFP^+^). (**i**) TDP-43 overexpressing progenitors fail to generate Tbr1^+^ neurons in the cortical plate. Scale bar in c and f = 25 µm. Dashed line marks the telencephalic vesicle lining. tTest ^*^P < 0.05, ^**^< 0.01, ^***^< 0.001.

Conversely, the moderate (2.4-fold) increase in wild type TDP-43 expression induced by IUE (Fig. 2a,b) resulted in an increase in active caspase-3 expressing cells and rapid apoptotic death of most neural progenitors *in vivo* (Fig. 2c,d). TDP-43 expression reduced not only the absolute number but also the proportion of Pax6^+^ cells remaining in the developing telencephalon (Fig. 2e). The few surviving TDP-43 overexpressing cells that were undergoing mitosis were displaced from their normal position at the ventricular lining (Fig. 2f,g). Furthermore, the differentiation of neural stem/progenitor cells into basal progenitors (Tbr2^+^) was reduced and generation of newborn neurons (Tbr1^+^) in the cortical plate was blocked by TDP-43 overexpression indicating cell death and impaired neuronal differentiation (Fig. 2h,i and Supplementary Fig. 3). These data suggested that overexpression of wild type TDP-43 in neural progenitors of the developing telencephalon is toxic, induces changes in cell proliferation and blocks neurogenesis.

### Mutant TDP-43^A315T^ induces p53-dependent cell death

Autosomal dominant mutations in human TDP-43 are sufficient to cause familial ALS and FTLD. Disease causing mutations occur predominantly in the C-terminal prion-domain of TDP-43^25^. The mechanisms of mutant TDP-43 induced toxicity remain unclear but are associated with an increase in TDP-43 protein levels^26^. We examined the effects of mutant TDP-43 expression in neural progenitors *in vivo*. Mutant human TDP-43^A315T^ (Fig. 2a), like wild type TDP-43, induced cell death and caspase-3 activation when expressed by IUE in neural progenitors *in vivo* (Fig. 2c). TDP-43^A315T^ expression resulted in a loss of Pax6^+^ progenitors in the ventricular zone and aberrant differentiation into basal progenitors (Tbr2^+^) (Fig. 2e,h). These data indicate that mutant TDP-43^A315T^ partially phenocopied the effects seen after overexpression of wild type TDP-43.

### TDP-43 induced cell death is p53-dependent

**Figure 3:**
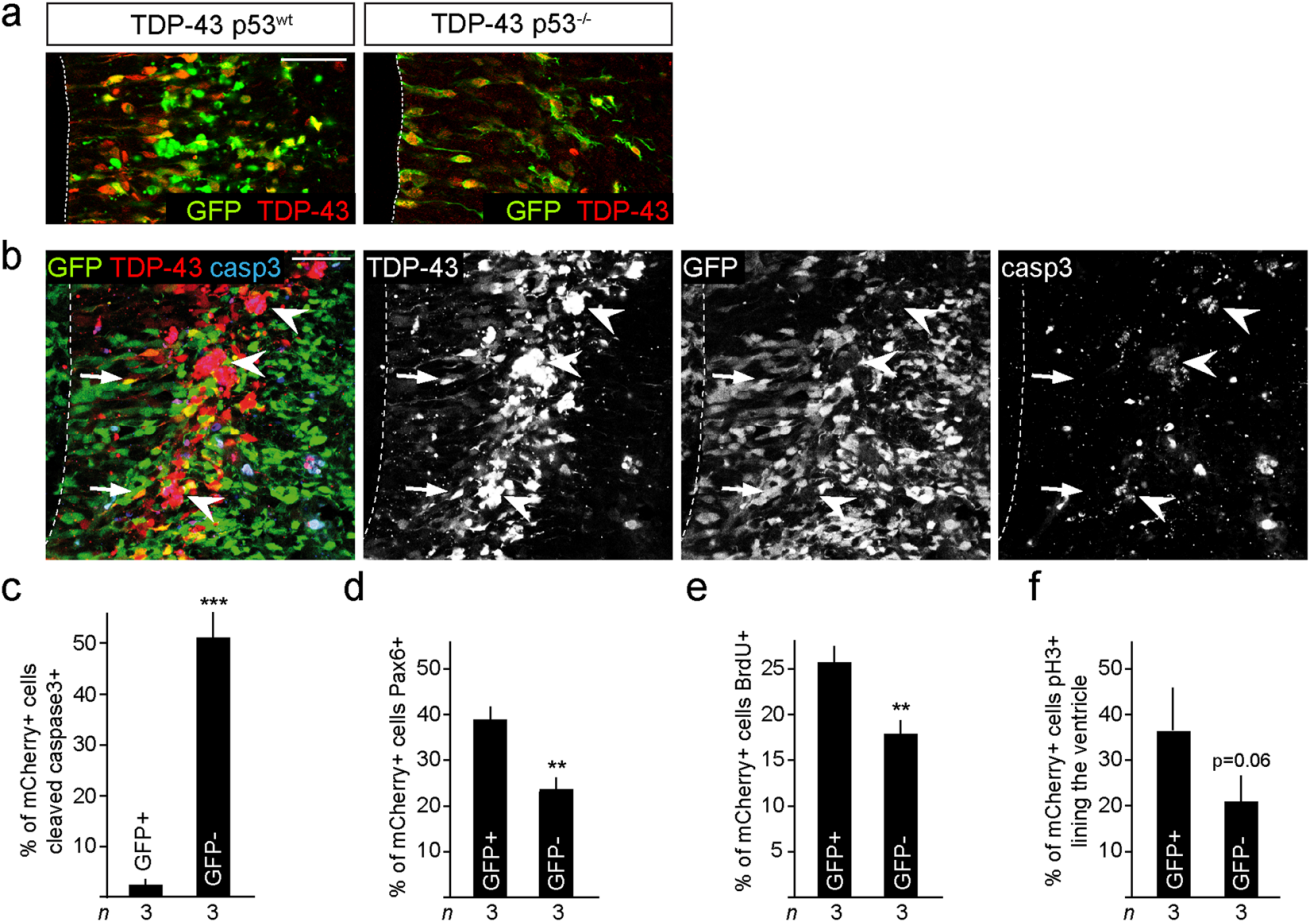
p53 deletion rescues TDP-43 overexpressing neural progenitors. (**a**) TDP-43 overexpression does not induce cell fragmentation or death of progenitors of p53^−/−^ embryos. p53^−/−^ TDP-43 expressing cells show a normal radial morphology in the ventricular zone and migrate to the subventricular zone. (**b**,**c**) Conditional deletion of floxed *Trp53* alleles by expression of Cre-recombinase (GFP^+^), (mCherry^+^GFP^+^p53^−/−^, arrows) reduces caspase-3 activation following expression of TDP-43 compared to none Cre-recombined cells (mCherry^+^GFP^-^p53^+/+^, arrowheads). Values are shown as the proportion of TDP-43 overexpressing cells (mCherry^+^). (**d–f**) *Trp53* ablated (GFP^+^), TDP-43 overexpressing (mCherry^+^) cells (mCherry^+^GFP^+^p53^−/−^, arrows) show rescue of Pax6^+^ (**d**), BrdU^+^ (**e**) and pH3^+^ expression (**f**) compared to none Cre-recombined *Trp53* wild type cells (mCherry^+^GFP^−^p53^+/+^). Values are shown as the proportion of transfected cells (mCherry^+^). Scale bar = 25 µm. Dashed line marks the telencephalic vesicle lining. tTest P = 0.06, ^**^< 0.01, ^***^< 0.001.

p53-dependent neuronal cell death occurs in several neurodegenerative diseases^27^. Given that TDP-43 induces cell death, the link between TDP-43 cleavage and caspases, and demonstration of elevated p53 in neurons of ALS patients^20,21^, we investigated whether TDP-43 induced apoptosis of neural progenitors cells depends on p53 activity. In contrast to expression in control (p53^wt^) embryos, IUE-mediated TDP-43 overexpression in p53-deficient embryos (p53^−/−^) did not disrupt neural progenitors in the ventricular zone or affect their morphology (Fig. 3a).

To separate potential cell autonomous and non-autonomous effects of TDP-43 overexpression, we performed a mosaic deletion of *Trp53* combined with IUE of TDP-43. We analyzed mice carrying *Hes5::CreER*^*T2*^ and *Rosa26* floxed STOP transcription GFP Cre reporter (*Rosa-CAG::GFP*) alleles, to lineage trace the Cre-expressing neural stem/progenitor cells and their progeny, which were either wild type or homozygous for floxed *Trp53* alleles. We induced deletion of *Trp53* from ventricular zone progenitors in embryos at e11 by Tamoxifen induction^28^ and expressed TDP-43 by IUE in these embryos at e13.5 (Supplementary Fig. 4a,b). Under these mosaic conditions, TDP-43 overexpressing cells that were wild type for *Trp53* (GFP^-^) underwent apoptosis (caspase-3) (Fig. 3b,c). Conversely, TDP-43 overexpressing cells where *Trp53* had been deleted (GFP^+^) were viable and showed a normal morphology (Fig. 3b,c). By this conditional approach, loss of p53 also rescued the reduction in Pax6^+^ progenitors and the proliferation defects (pH3^+^ and BrdU labeling) induced by TDP-43 overexpression (Fig. 3d–f). This suggested that the cell death caused by TDP-43 is a p53-dependent cell autonomous response and not the result of disruption of the neural progenitor zones in the developing telencephalon.

**Figure 4:**
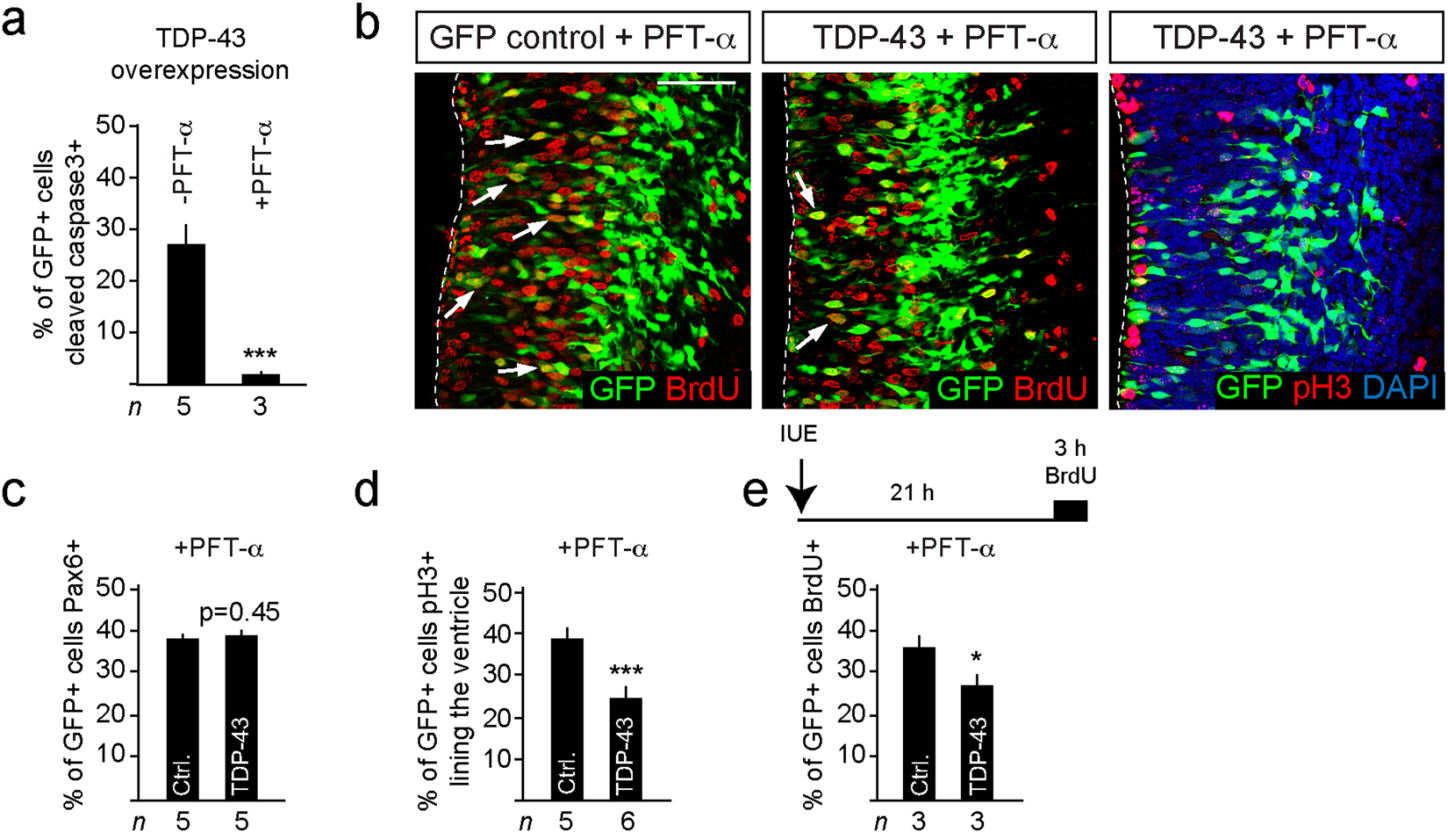
p53 inhibition with PFT-α rescues TDP-43 mediated apoptosis and integrity but not cell cycle defects. **(a**) TDP-43 induced caspase-3 activity and apoptosis is rescued by blocking p53 with PFT-α. (**b**) Inhibition of p53 with PFT-α *in utero* rescues TDP-43 induced apoptosis but does not reverse cell cycle defects. Arrows indicate BrdU^+^ transfected cells (GFP^+^). (**c**) Inhibition of p53 with PFT-α rescues TDP-43 expressing Pax6^+^ progenitors in embryos. (**d**) The proportion of TDP-43 overexpressing cells (GFP^+^) at the ventricular lining and expressing pH3^+^ is reduced and not rescued by PFT-α compared to control transfected cells (Ctrl.). Values are shown as the proportion of transfected cells (GFP^+^). (**e**) The proportion of TDP-43 overexpressing cells (GFP^+^) in the VZ that are BrdU labeled is not rescued by PFT-α compared to control cells (Ctrl.). Values are shown as the proportion of transfected cells (GFP^+^). Scale bars = 25 µm. Dashed line marks the telencephalic vesicle lining. tTest ^*^P < 0.05, ^***^< 0.001.

Similarly, injection of the pharmacological inhibitor of p53, Pifithrin-α (PFT-α)^29,30^, into p53^wt^ mothers rescued TDP-43 overexpressing cells and blocked caspase-3 activation (Fig. 4a,b). We analyzed PFT-α rescued cells in greater detail and found that the number and integrity of ventricular zone progenitors (Pax6^+^) and basal progenitors (Tbr2^+^) were comparable to controls (Fig. 4b,c and data not shown). Mitotically active cells (pH3^+^) were decreased upon TDP-43 expression, but this was not rescued by inhibition of p53 **(**Fig. 4d). Furthermore, PFT-α treatment did not induce a change in the number of pH3^+^ cells lining the ventricle in control (35.6 +/− 3.1 versus 39.0 +/− 1.8%) or TDP-43 overexpressing embryos (18.1 +/− 2.8% versus 23.4 +/− 3.0) (compare Figs 2g and 4d). Reduced proliferation in the ventricular zone following TDP-43 overexpression was also supported by BrdU incorporation experiments with less labeled cells in the TDP-43 overexpressing embryos even after PFT-α treatment (Fig. 4e). These findings are suggestive of two independent mechanisms of action for TDP-43 overexpression, one triggering p53-dependent apoptosis and a second perturbing cell cycle dynamics that likely does not involve p53.

### RNA recognition motif 1 of TDP-43 is critical for toxicity

The underlying mechanisms through which TDP-43 induces cell death are unclear. Both full length as well as truncated and C-terminal fragments of TDP-43 accumulate in aggregates during neurodegeneration^31^. TDP-43 binds thousands of RNAs, including its own, regulating many aspects of RNA biogenesis, stability, splicing and transport^15,16,32^. The RNA recognition motif 1 (RRM1) of TDP-43 is necessary and sufficient for RNA binding^6,33^. We addressed whether RNA binding is required for TDP-43 induced toxicity in neural progenitors by expressing a mutant form where the RRM1 had been deleted (TDP-43^∆RRM1^) (Supplementary Fig. 5a,b). Unlike TDP-43 and TDP-43^A315T^, expression of TDP-43^∆RRM1^ in neural progenitors did not result in obvious signs of cell death *in vivo* (Supplementary Fig. 5c). The number of ventricular zone progenitors (Pax6^+^) and basal progenitors (Tbr2^+^) were comparable to controls following TDP-43^∆RRM1^ expression (Supplementary Fig. 5d–f). This indicated that functional RNA binding of TDP-43 is crucial in the cell death response and aberrant neurogenesis induced by TDP-43.

### TDP-43 and TDP-43^A315T^ induce p53 targets and proapoptotic gene expression

**Figure 5:**
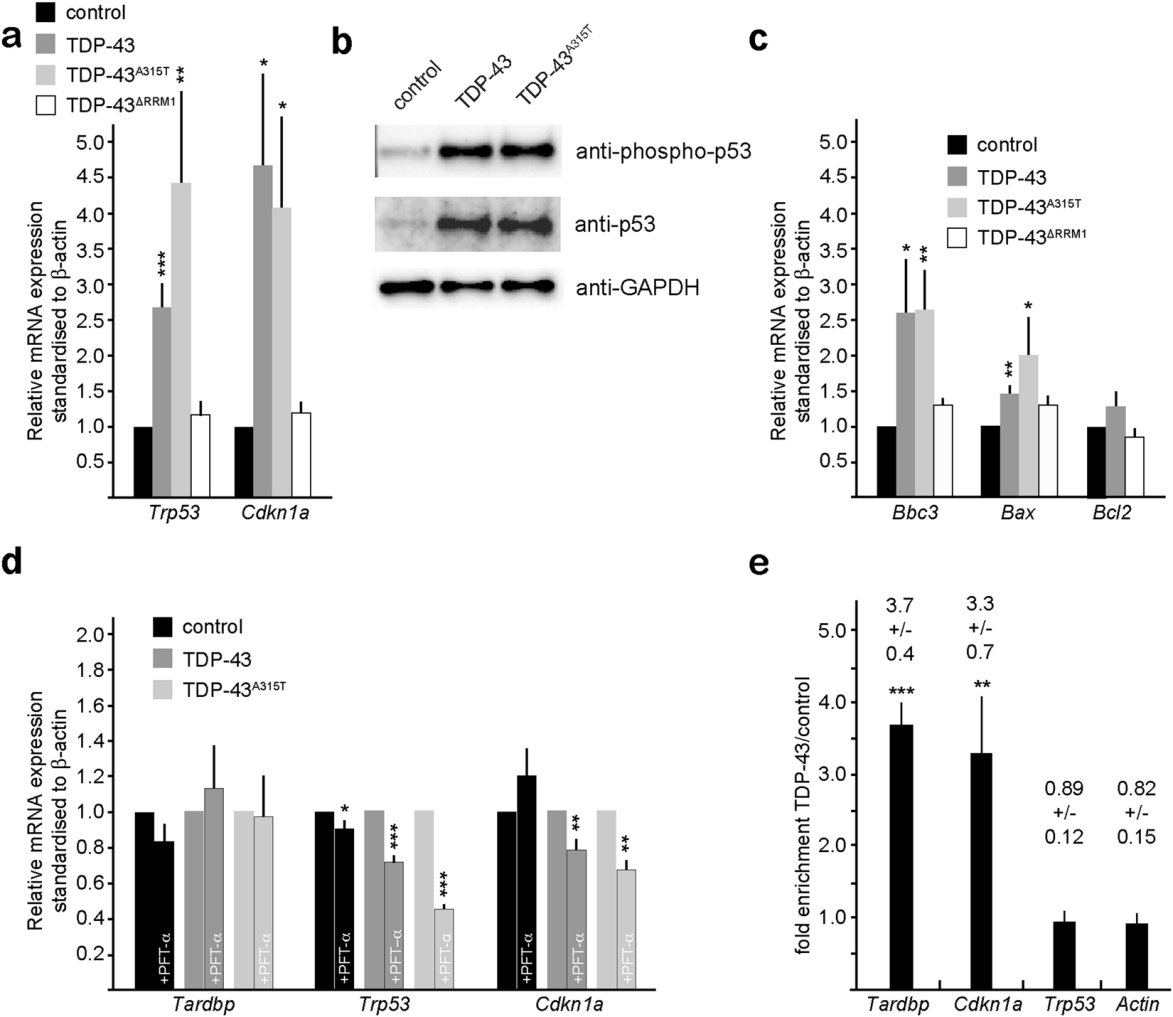
TDP-43 regulates proapototic BH3-only, Trp53 and Cdkn1a mRNA expression. (**a**) TDP-43 and TDP-43^A315T^ expression in neural progenitors increases *Trp53* and *Cdkn1a* mRNA levels, analyzed by quantitative RT-PCR analysis. TDP-43^∆RRM1^expression does not affect *Trp53* and *Cdkn1a* mRNA levels. (**b**) TDP-43 and TDP-43^A315T^ expression in neural progenitors results in increased level of activated, phosphorylated p53 protein and total p53 protein. (**c**) TDP-43 and TDP-43^A315T^ expression in neural progenitors increases mRNA levels of the proapoptotic BH3-only proteins *Bbc3* (PUMA) and *Bax*, but not the antiapoptotic factor *Bcl2*. TDP-43^∆RRM1^expression does not affect *Bbc3*, *Bax* or *Bcl-2* mRNA levels. (**d**) Pharmacological inhibition of p53 reduces TDP-43 and TDP-43^A315T^ induced *Trp53* and *Cdkn1a* mRNA levels but does not affect endogenous *Tardbp* mRNA expression, analyzed by quantitative RT-PCR analysis. Values are shown as relative to non-treated, standardized to *β-actin*. (**e**) TDP-43 binds endogenous *Tardbp* and *Cdkn1a* but not *Trp53* mRNAs. Quantitative RT-PCR analysis of *Tardbp*, *Cdkn1a*, *Trp53* and *β*-actin transcripts CLIPed together with TDP-43 from neural progenitors. Values are fold enrichment over control CLIPed (flag-GFP) transcripts. Statistical analysis of CLIPed products corrected relative to input RNA concentrations compared to flag-GFP CLIPed samples. Agarose gel analysis of the amplicons is shown in Supplementary Fig. 6a.

TDP-43 and TDP-43^A315T^ induced cell death was blocked by PFT-α. TDP-43 and TDP-43^A315T^ increased *Trp53* mRNA and p53 protein levels consistent with an activation of a stress response (Fig. 5a,b). In addition to the increase in total p53 levels, the phosphorylated and active form of p53 was also increased substantially following TDP-43 and TDP-43^A315T^ expression (Fig. 5b). p53 regulates proapoptotic gene expression including *Bbc3* (PUMA) and *Bax* as well as cell cycle regulators including *Cdkn1a*. Consistent with an activation of p53, TDP-43 and TDP-43^A315T^ induced *Bbc3* and *Bax* expression in neural progenitors but not the antiapoptotic gene *Bcl2* (Fig. 5c). In contrast, TDP-43^∆RRM1^ overexpression did not affect p53 or proapoptotic gene expression (Fig. 5a,c). The balance between pro- and antiapoptotic genes regulates entry into the apoptotic pathway by stimulating mitochondrial permeability, the release of cytochrome C, and activation of caspase-9 and caspase-3^34^. Bbc3 binds Bcl2 releasing the proapoptotic BH3-only proteins Bax and Bak. Hence, Bbc3 induced by TDP-43 via p53 could bind and inhibit Bcl2, and the concomitant enhanced Bax expression would result in increased cell death.

*Cdkn1a* is a direct transcriptional target of p53. TDP-43 and TDP-43^A315T^ but not TDP-43^∆RRM1^ induced *Cdkn1a* expression (Fig. 5a). PFT-α treatment did not affect the expression of the endogenous *Tardbp* but significantly inhibited TDP-43 and TDP-43^A315T^ induced *Trp53* and *Cdkn1a* expression (Fig. 5d). Hence, overexpression of TDP-43 and a mutant form, TDP-43^A315T^, result in activation of multiple p53 target genes and this requires the RRM1 domain.

### Cdkn1a mRNA associates with TDP-43

As TDP-43 reduced cell cycle, and induced *Cdkn1a* expression (Figs 2–5**)**, we examined the cell cycle defects in more detail. Therefore, we assessed a potential TDP-43 regulation of *Trp53* and *Cdkn1a* by CLIP (cross-linked RNA immunoprecipitation). In accordance with previous findings^15,16,32,35^, TDP-43 bound and decreased endogenous *Tardbp* mRNA levels (Fig. 5e and Supplementary Fig. 6a,b). In addition, *Cdkn1a* but not *Trp53* mRNA was CLIPed together with TDP-43 (Fig. 5e and Supplementary Fig. 6a) and expression of TDP-43 and TDP-43^A315T^ translated into an increase in Cdkn1a protein in N2A cells (Supplementary Fig. 6c). Hence, although PFT-α reduced *Trp53* and *Cdkn1a* mRNA expression in neural progenitors *in vitro* (Fig. 5d), and rescued cell death, Cdkn1a protein levels still increased in response to TDP-43, likely as a result of regulation of Cdkn1a at the post-transcriptional level. We did not observe an interaction between TDP-43 and p53 or Cdkn1a at the protein level (Supplementary Fig. 6d). Forced expression of Cdkn1a by IUE resulted in exit of progenitor cells from the ventricular zone and their exit from the cell cycle but, unlike TDP-43 overexpression, did not induce cell death or precocious differentiation (Supplementary Fig. 7a–d). Interestingly, we observed a reciprocal down regulation of TDP-43 mRNA and protein expression following overexpression of Cdkn1a (Supplementary Fig. 6b and Fig. 2b). Hence, TDP-43 regulates Cdkn1a expression that potentially contributes to the changes in cell cycle and explains the insensitivity of these effects to p53 inhibition.

### p53 inhibition partially rescues TDP-43^A315T^ embryos

Homozygous mice expressing TDP-43^A315T^ from the *Tardbp* locus (*Tardbp*^315*/315*^) die *in utero* (Supplementary Fig. 7a)^36^. The cause of death is unclear but post-implantation e6.5 mutant embryos were developmentally retarded, showed hemorrhage around the primitive endoderm and limited trophectodermal expansion and infiltration. At e9.5 most *Tardbp*^*315/*315^ embryos were dead and the few remaining mutants were severely developmentally delayed and died soon thereafter (Supplementary Fig. 8a). We treated pregnant *Tardbp*^*wt/315*^ females from *Tardbp*^*wt/315*^ inter-crosses with PFT-α every day after day 3.5 post-coitum. Inhibition of p53 *in utero* rescued *Tardbp*^*315/315*^ embryos past e7.5 to Mendelian ratios up to e14.5 (Supplementary Fig. 8b,c). The PFT-α rescued mice looked phenotypically normal although slightly smaller than age-matched siblings. Brain morphology and development did not show obvious defects and proliferation within the brain of e14.5 *Tardbp*^*315/315*^ embryos was indistinguishable from wild type siblings (Supplementary Fig. 8d,e). In order to address whether loss of p53 was able to rescue *Tardbp*^*315/315*^ mice to birth, we inter-crossed *Tardbp*^*wt/315*^ and Trp53^−/−^ mice to generate *Tardbp*^*315/315*^, p53^−/−^ animals. However, after analysis of >100 offspring, we were unable to find *Tardbp*^*315/315*^ mice in multiple litters from different parents. Hence, inhibition of p53 with PFT-α rescued early embryonic death of *Tardbp*^*315/315*^ mutant mice but inhibition of p53 function is unable to rescue *Tardbp*^*315/315*^ embryos to birth.

### TDP-43 induced cell death of human iPS-derived cortical neurons can be rescued by blocking p53

**Figure 6:**
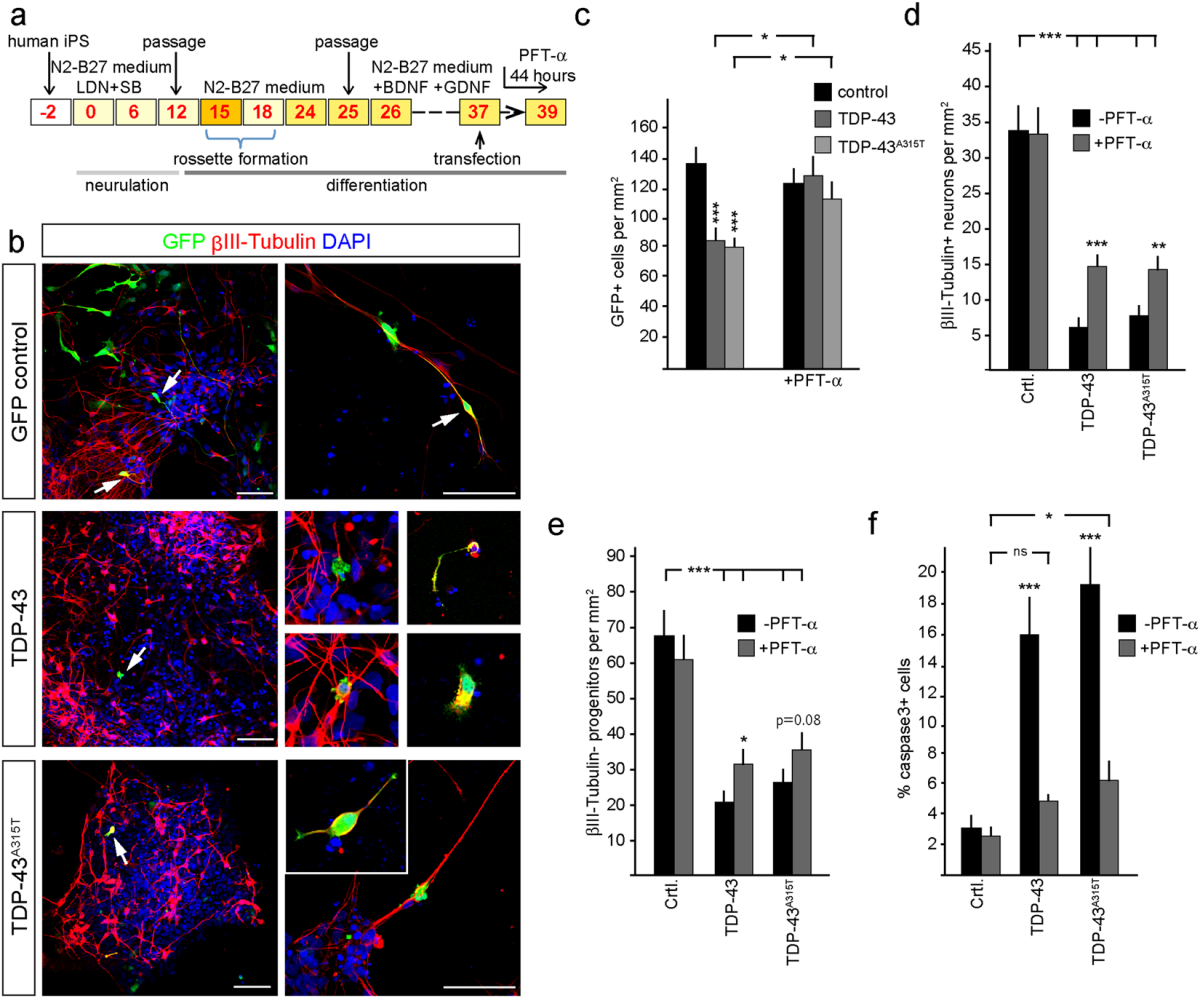
TDP-43 and human mutant TDP-43^A315T^ toxicity in human iPS-derived neurons is rescued by inhibition of p53. (**a**) Scheme of the human iPS cortical neuron differentiation profile and analysis of TDP-43 overexpression. Control human iPS cells were neuralized from day 0 to day 12 in N2-B27 Medium + LDN and SB and passaged on day 15 and day 25 and differentiated in N2 medium minus B27 (N2-B27) for 39 days. TDP-43 and TDP-43^A315T^ expression constructs were transfected on day 37. (**b**) Transfected GFP-expressing human iPS cells have neuronal and progenitor morphologies. TDP-43 and TDP-43^A315T^ cells are reduced and show stunted morphologies and cellular fragmentation compared to controls (arrows). (**c**) Expression of TDP-43 and TDP-43^A315T^ reduces the number of iPS-derived cells within the cultures. Treatment with PFT-α for 44 hours significantly rescues the number of transfected cells to control (GFP) levels. Together with the increased expression of activated caspase-3 these findings confirm that TDP-43 and TDP-43^A315T^ are toxic and rapidly induce apoptotic cell death that is dependent upon p53. (**d**,**e**) TDP-43 and TDP-43^A315T^ expression result in a reduction of iPS-derived neurons (βIII-Tubulin^+^) and progenitors cells (βIII-Tubulin^−^). PFT-α treatment of the cultures for 44 hours prior to analysis partially rescued the TDP-43 and TDP-43^A315T^ induced loss of neurons and progenitors. (**f**) TDP-43 and TDP-43^A315T^ expressing human neurons activate caspase-3 and die by apoptosis. Inhibition of p53 with PFT-α for 44 hours rescues cell death. Scale bar = 25 µm. tTest ^*^P < 0.05, ^**^< 0.01, ^***^< 0.001, ns not significant.

TDP-43 and TDP-43^A315T^ expression induces p53-dependent apoptosis in the developing mouse telencephalon. In order to assess the effects of accumulated TDP-43 on human cortical cells, we differentiated human iPS cells to cortical progenitors and neurons for 39 days (Fig. 6a)^37^. Expression of wild type TDP-43 and mutant TDP-43^A315T^ at day 37 caused a loss of human iPS-derived cortical cells within 48 hours including neurons and progenitors (Fig. 6b–d). The surviving TDP-43 overexpressing cells had stunted morphologies and pyknotic nuclei (Fig. 6b). Treatment of TDP-43 and TDP-43^A315T^ expressing human iPS-derived cortical cultures with the PFT-α for the last 44 hours of culture rescued cell number and partially rescued the loss of neurons and progenitors and reduced the number of active caspase-3 expressing cells (Fig. 6c–f). Hence, TDP-43 accumulation in human cortical neurons can also induce p53-dependent neuronal cell death.

### TDP-43 induces increased proapoptotic gene expression in human iPS-derived cortical cells

**Figure 7:**
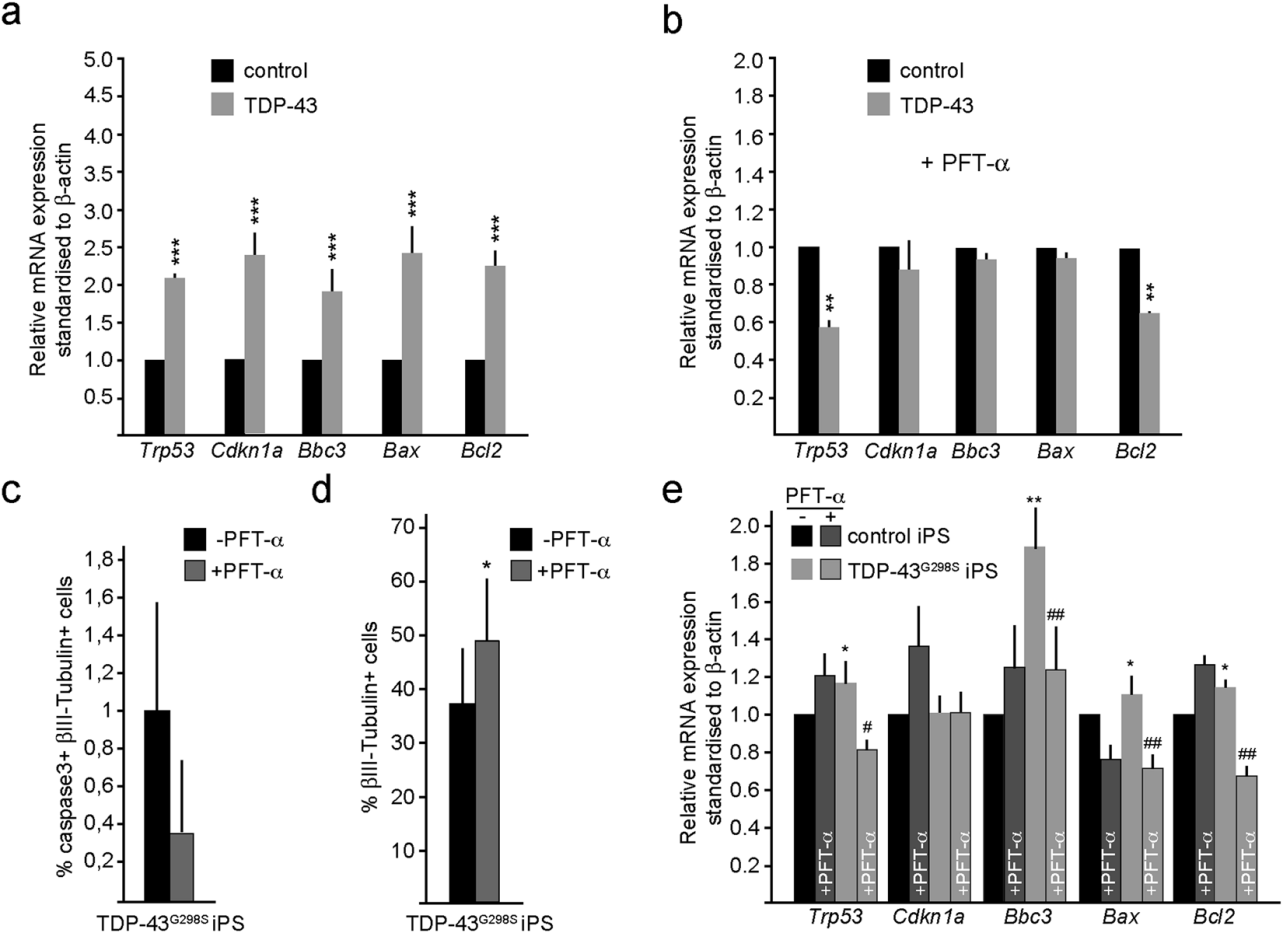
Proapoptotic gene expression is increased in human iPS-derived cortical cells and TDP-43^G298S^ mutant iPS derived cortical cultures. (**a**) TDP-43/GFP expression in human iPS followed by fluorescent assisted cell sorting after 48 hours. TDP-43 expression in human iPS cortical neuron increases mRNA levels of the proapoptotic genes *Trp53*, *Cdkn1a*, *Bbc3* (PUMA), *Bax* and *Bcl2*. (**b**) TDP-43/GFP expression in human iPS followed by sorting after 48 hours. p53 inhibition by PFT-α treatment for 44 hours prior to sorting rescues TDP-43 induced increase of proapoptotic genes in human iPS compared to control. **(c**) TDP-43^G298S^ iPS-derived cells show reduced numbers of activated caspase-3^+^ cells upon PFT-α treatment for 44 hours. (**d**) TDP-43^G298S^ iPS cells and control iPS cells show reduced numbers of βIII-Tubulin^+^ cells upon PFT-α treatment for 44 hours. (**e**) TDP-43^G298S^ iPS cells display increases mRNA levels of proapoptotic genes *p53*, *Bbc3* (PUMA), *Bax* and *Bcl2* upon PFT-α treatment for 44 hours. In comparison mRNA levels of the very genes in Control iPS cells are unchanged. tTest ^*^P < 0.05, ^**^< 0.01, ^***^<0.001 relative to control cells. ^#^P < 0.05, ^##^P < 0.01 relative to cells not treated with PFT-α.

As TDP-43 overexpression in human iPS-derived cortical cells induced cell death, we examined whether this involved increased expression of the proapototic genes *BBC3*, *BAX* and the antiapototic gene *BCL2*. We transfected iPS-derived cortical cells at day 37 of differentiation, sorted the TDP-43 overexpressing human cortical cells (GFP^+^) after 48 hours and examined gene expression by qRT-PCR. In agreement with our findings in the mouse telencephalon, overexpression of TDP-43 in human cells significantly increased the expression of *TRP53*, *CDKN1A*, *BBC3*, and *BAX* but also *BCL2* (Fig. 7a). We examined whether the increase in proapoptotic gene expression in response to TDP-43 overexpression was dependent upon p53 activity by treating the cells for the last 44 hours of culture with PFT-α. Blocking p53 activity prevented the increase in *TRP53*, *CDKN1A*, *BBC3*, *BAX* and *BCL2* expression above the levels seen in PFT-α treated control cultures, suggesting that also in human cortical cells, the expression of TDP-43 induces a p53-dependent increase in proapoptotic genes that may contribute to the observed cell death (Fig. 7b).

TDP-43 mutations are associated with neurodegenerative disease including FTLD and ALS. We used patient-derived *TDP-43*^*G298S*^ mutant iPS cells^38^ and differentiated them into cortical neurons using the same protocol used for differentiation of the control human iPS cells (Fig. 6a). These cortical cultures contained activated caspase-3^+^ neurons and treatment with PFT-α for 44 hours reduced the number of apoptotic neurons suggesting p53 activity in these cultures (Fig. 7c). In agreement with p53-mediated neuronal cell death, PFT-α treatment significantly increased the number of neurons derived from TDP-43^G298S^ patient iPS cells (Fig. 7d). Therefore, we examined the expression levels of *TRP53*, *CDKN1A*, *BBC3*, *BAX* and *BCL2* in the *TDP-43*^*G298S*^ mutant iPS-derived cortical cultures. *Trp53* expression was significantly higher in the *TDP-43*^*G298S*^-derived cells compared to control. In support of an increased p53 activity, the expression of *BBC3*, *BAX* and *BCL2* were all elevated in the *TDP-43*^*G298S*^ iPS cortical neuron cultures. In addition, treatment of the iPS-derived cultures with PFT-α to block p53, significantly reduced expression of *BBC3*, *BAX* and *BCL2* in *TDP-43*^*G298S*^ but not by control iPS-derived cortical neurons (Fig. 7e).

## Discussion

Our findings identify activation of proapototic gene expression as a mechanism through which TDP-43 accumulation and mutations can induce cell death in murine neural progenitors *in vivo* and human cortical neurons *in vitro*. This effect was rescued by blocking or depleting p53. p53 is induced by cellular stress and recent findings indicate that TDP-43 accumulation, as seen in neurodegenerative diseases including ALS and FTLD, induces cellular stress response^24,39–41^. Thus, it is possible that induced p53 in our paradigms is also a result of cellular stress caused by the accumulation of TDP-43. In addition, TDP-43 induces phosphorylation of Eif2α, which results in a general inhibition of translation^30^. Eif2α phosphorylation induces translation of ATF4 (activating transcription factor 4), which enhances expression of ATF3 and CHOP (CCAAT/enhancer-binding protein homologous protein). CHOP inhibits Bcl2 functions at the protein level and induces Bim to enhance cell death through activation of Bax. In agreement, we found that neither TDP-43 nor TDP-43^A315T^ induce *Bcl2* expression in mouse cells, whereas *Bax* is increased. However, it is conceivable that Eif2α inhibition of antiapoptotic BH3-only proteins and the p53-induced proapoptotic gene (*Bbc3* and *Bax*) expression synergize to promote cell death in TDP-43 mutant cells. Hence, TDP-43 seems to activate a bifurcated pathway to control BH3-only protein-mediated apoptosis.

Increase of TDP-43 and TDP-43^A315T^ in cortical neurons derived from human iPS cells also resulted in an apoptosis phenotype. In concordance with the overexpression in mouse cells, we observed a strong increase in *TRP53*, *CDKN1A*, *BBC3* and *BAX* in the human cortical neural cells *in vitro*. However, *BCL2* was also increased in the human cortical neurons in contrast to mouse neural progenitor cells. This opposing result might stem from differences in pathway regulation between species. However, inhibition of p53 by PFT-α rescued the TDP-43 mediated increase in the proapoptotic genes in mouse neural progenitor cells and human cortical neurons.

Besides the TDP-43 mediated increase in p53, we also observed a strong increase in *Cdkn1a* mRNA levels. Concomitant, we observed that TDP-43 overexpression induced a block in cell cycle progression in mouse neural progenitors *in vivo*. *Cdkn1a* is activated by p53, however, we additionally show an association of TDP-43 to *Cdkn1a* mRNA, which suggests a regulation of Cdkn1a expression by TDP-43. This is supported by the observation that blocked cell cycle progression could not be rescued by p53 inhibition. Thus, TDP-43 expression not only induces apoptosis but also induces a cell cycle block, probably at G2/M phase transition through cyclin dependent kinase inhibition via Cdkn1a, which in turn could stimulate cell death. Due to the fact that we performed the CLIP experiments with PFA cross-linking, we cannot be sure that TDP-43 binds directly to *Cdkn1a* mRNA. Confirmation of direct binding of TDP-43 to *Cdk1a* mRNA will require further analysis. Previously, it has been reported that TDP-43 overexpression induces G2/M arrest in HeLa cells^42^. It was shown that the effect was partially dependent on p53 but apoptosis could not be rescued by p53-inhibition. These partially contradictory results may be explained by the differences in cells used in the experiments. HeLa cells have an altered cell cycle regulation and might not respond in the fashion as neural progenitors and neurons to inhibition of p53.

Many lines of transgenic mice expressing wild type or mutant forms of TDP-43 have been analyzed in the last years, all of which show early mortality. We employed homozygous mice expressing TDP-43^A315T^ and partially rescued the animals by p53 inhibition. Similar rescue experiments have been preformed in *hSOD1*^*G82A*^ transgenic mice. Interestingly, p53 deletion did not rescue the early lethality in these *hSOD1*^*G82A*^ animals, which indicates a p53-independent mechanism, possibly involving death receptors^34^. This is supported by findings that death receptor 6 (DR6) levels are elevated in *hSOD1*^*G82A*^ transgenic mice^43^. In addition, treatment with blocking antibodies against DR6 increased motor neuron survival and, therefore, provided a neuroprotective effect in *hSOD1*^*G82A*^ expressing animals. The partial rescue of TDP-43^A315T^ embryos indicates probable involvement of additional factors triggering lethality of these animals. However, this remains to be addressed in the future.

We also investigated how loss of TDP-43 affects cell survival and differentiation of neural stem/progenitor cells. In striking contrast to the overexpression experiments, we did not observe signs of apoptosis upon KD of TDP-43 *in vivo*. Although TDP-43 is indispensable for early development^3^, it does not seem to have a crucial function in neural progenitor maintenance in the brain at the time points we analyzed. This might be due to the fact that we only achieved a 70% reduction in TDP-43 levels at the protein level. However, we did observe a significant increase in the number of proliferating cells in the *in vivo* KD experiment. *Cdkn1a* KD induces proliferation by promoting S-phase entry^44^. We hypothesize that loss of TDP-43 results in a reduction in Cdkn1a, which in turn activates proliferation, reduces cell cycle exit and delays differentiation.

In conclusion, we show that TDP-43 accumulation in mouse neural progenitors *in vivo* as well as in human cortical progenitors and neurons and in TDP-43^G298S^ mutant iPS-derived cortical cultures induces p53-dependent apoptosis. This is associated with up regulation of apoptotic genes including *Bbc3* and *Bax*. We did not observe a direct interaction between TDP-43 and p53 on the protein or mRNA levels. It therefore remains elusive how TDP-43 activates p53 expression but this is likely, at least in part, to be indirect via induction of cellular stress response^30^. Future work should address this as well as how the multiple pathways downstream of TDP-43 contribute to cell death in disease.

## Methods

### Animals and animal husbandry

*Trp53*^*tm1Tyj*^, *Trp53*^*tm1Brn*^, *TDP-43*^*A315T*^, *Rosa-CAG::GFP* and *Hes5::CreER*^*T2*^ mice have been described elsewhere^28,36,45–47^. Mice were maintained on a 12-hour day/night cycle with adequate food and water under SPF conditions. All methods were carried out in accordance with guidelines and regulations of Max-Planck Institutional and German Federal regulations and under license numbers H-05/01, 0-06/02, G-09/18, G-09/19, G-08/26 (Ethical Commission Freiburg, Germany) and 2437 and 2438 (Veterinary commission Basel). All experimental protocols were approved by Max-Planck Institutional and German Federal regulations and under license numbers H-05/01, 0-06/02, G-09/18, G-09/19, G-08/26 (Ethical Commission Freiburg, Germany) and 2437 and 2438 (Veterinary commission Basel). The day of vaginal plug was counted as embryonic day 0.5 (e0.5).

### Tamoxifen, BrdU and PFT-α administration

*Trp53*^*tm1Brn*^/*Hes5::CreER*^*T2*^/*Rosa-CAG::GFP* mice were given 2 mg Tamoxifen by gavage at e11.5 to induce recombination. Stock solutions of Tamoxifen (Sigma) were prepared at a concentration of 20 mg/ml in corn oil (Sigma). Bromodesoxyuridine (BrdU, Sigma) was administered to the adult animals via a single intraperitoneal injection (50 mg/kg body weight). PFT-α (stock solution 20 mM in DMSO) was injected intraperitoneal into pregnant mice (2.2 mg/kg).

### *In utero* electroporation for overexpression in neural stem/progenitors *in vivo*

Female C57BL/6 J and *Trp53*^*tm1Brn*^ mice were used for the *in utero* expression analyses at 13.5 days after detection of vaginal plug. DNA constructs were injected into the forebrain telencephalic vesicles of the embryonic mice using a microinjector (Pneumatic Pico Pump, WPI Rnage) and pulled Borosilicate glass capillaries (Kwick-Fil^TM^). The capillaries were pulled in a micropipette puller (Sutter Instrument Co.) and sharpened using a capillary sharpener (Bachofer). The capillaries were backend-loaded with 10 μl of the plasmid. Plasmid stocks were prepared under endotoxin free conditions and suspended in sterile phosphate buffered saline (PBS) at a concentration of 3 μg/μl. Fast green contrast dye (10%) was added to the plasmids to visualize the targeted area of the telencephalon. The overexpression or shRNA knockdown vectors were electroporated in a molecular ratio of 3:1 with the transfection reporter vector (*pCAGGS::eGFP*). The pregnant female mice were administered analgesic (Temgesic; 0.8 mg/kg) by intraperitoneal injection and anesthetized with 1–2% isoflurane (Baxter) in a constant flow of O_2,_ secured on a heated operating table. Body temperature was monitored continually. The fur was removed from the abdomen using depilation cream. Throughout the operation, the peritoneal cavity was moistened with sterile Hank’s buffered saline solution (HBSS). The uterine horns containing the embryos (e13.5) were manipulated under sterile conditions by hand. A cold light source was used to illuminate the embryos. 1–2 μl of DNA solution were injected into the telencephalic vesicles of each embryo. The embryos were electroporated (Electro Square Pavator™, BTX^®^ Harvard Apparatus) with 10 pulses of 40 V and a pulse length of 50 ms at 950 ms intervals. The anode of the electrode was oriented toward the injected side. After injection and electroporation, the uterus was returned to the abdomen, the muscle and the skin sutured and the females allowed to recover under a heating lamp with constant observation. Operated females had free postoperative access to analgesic (Temgesic; 0.8 mg/kg) in sterile agar. The animals were sacrificed after a defined time and the embryos isolated and prepared for sectioning.

### Expression plasmids and constructs

Full-length coding region cDNAs for GFP, Cdkn1a, human TDP-43, human TDP-43^A315T^ and human TDP-43^∆RRM1^ were subcloned into *pCAGGS* expression vectors with a beta-globin 3-prime untranslated and polyadenylation sequence. *pSuper-shRenilla* as well as *pSuper-shTDP-43* were cloned according to the manufacturer’s instructions (Oligoengine). *p3X-FLAG-myc-CMV*^*TM*^*-26* expression construct was obtained from Sigma and *p3X-TDP-43-flag* was cloned using Not1 and Xba1 restriction sites (Primers: fwd 5′- TTCTGCGGCCGCCACCATGTCTGAATATATTCG-3′ rev 5′-CTTTCTAGACTACATTCCCCAGCCAGAAG-3′).

### *In situ* RNA hybridization

For *in situ* RNA hybridization a digoxigenin- (DIG)-labeled antisense RNA probe was generated for mouse *Tardbp* RNA (amplified from mouse cDNA using primers: fwd 5′-ATTCCTTCCCGTCTGTGCTT-3′ rev 5′-TGCTTAGGTTCAGCATTGGA-3′) using the procedures described previously. Expression was detected by colorimetric reaction using NBT (nitroblue tetrazolium chloride) and BCIP (5-bromo-4-chloro-3-indolylphosphate p-toluidine) as reaction substrates and images taken using an Axioplan microscope (Zeiss) with an Axiocam CCD camera (Zeiss).

### Quantitative real-time PCR analysis of gene expression

For RNA isolation, cells were lysed directly in Trizol (Invitrogen) reagent. RNA was prepared according to the manufacturer’s instructions. 1 μg of total RNA was used for cDNA synthesis by Oligo-dT priming and BioScript (Bioline). For quantitative RT-PCR the SensiMix SYBR kit (Bioline) was used following the manufacturer’s instructions (see Supplemental Materials and Methods for detailed description of primers) The reaction was run in a Rotor-Gene^TM^ 6000 Real-time PCR machine (Corbett) and analyzed using Rotor-gene 6000 series software 1.7. β-actin and GAPDH mRNA levels were measured as endogenous controls and for quantification.

### Cross-linked RNA immunoprecipitation

The detailed protocol can be found at http://www.bio-protocol.org/e398. In summary, N2A cells were transfected using Lipofectamin2000 (Invitrogen) according to manufacturer’s instructions with *p3X-flag-GFP* or *p3X-flag-TDP-43* and trypsinized after 48 hours. The cells were then fixed in 3% formaldehyde in PBS for 10 minutes and lysed by sonication (10 pulses for 10 seconds). Immunoprecipitation was performed overnight at 4 °C using anti-Flag M2 Affinity Gel (Sigma-Aldrich). After collection by centrifugation at 2000g and washing 3–4 times with lysis buffer the complexes were reverse cross-linked and RNA extracted using Trizol reagent (Invitrogen) according to the manufacturers instructions. Isolated RNA was treated with RNase-free DNaseI (Roche) to remove any genomic DNA contamination. First strand cDNA was generated using BioScript (Bioline) and random hexamer primers followed by real-time PCR using SensiMix SYBR Kit (Bioline).

### Immunoprecipitation and Western blotting

For immunoprecipitation, N2A cells were transfected with expression plasmids (*p3X-flag-TDP-43* or *p3X-flag-GFP* as control) and cells were harvested after 24 hours. Cells were lysed and 1/10 of the lysate was used as input control. The remaining lysate was added to pre-blocked beads coupled to anti-flag antibody (ANTI-FLAG^®^ M2 Affinity Gel) or to un-coupled beads (Sepharose-G beads) and incubated at 4 °C. Thereafter the beads with the bound proteins were washed and the samples were incubated for 1 hour at 70 °C to release the bound proteins. The supernatant containing the immunoprecipitated proteins was then analyzed on a SDS-gel. For low molecular weight proteins like TDP-43, 15% separation gels were used according to the manufacturer’s instruction. 20–40 μg proteins with sample loading buffer were denatured and loaded on the gel. Proteins were separated and after blotted onto a PVDF membrane. To detect proteins the membrane was incubated with specific antibodies after blocking with blocking buffer for 1 hour (see Supplemental Tables for detailed description of antibodies). The membrane was thereafter incubated with a secondary antibody coupled to horse reddish peroxidase and the antibody was detected using Amersham™ ECL™ Western Blotting Detection reagent.

### Immunohistochemistry and immunostaining

Embryos were harvested and the brains fixed in 4% paraformaldehyde (PFA) solution in 0.1 M phosphate buffer (PB) overnight. Brains were cryoprotected in a 30% sucrose solution in 0.1 M PB for 24 hours, embedded and frozen over dry ice in OCT (TissueTEK). Horizontal sections (20 μm) were collected on Superfrost glass slides (Thermo Scientific), and stored at −20 °C until use. For immunostaining, sections were incubated overnight at 4 °C with the primary antibody diluted in blocking solution of 2% normal donkey serum (Jackson ImmunoResearch), 0.3% Triton X-100 in 0.1 M PB. Sections were washed three times in PBS and incubated at room temperature for 3 hours with the corresponding secondary antibodies in blocking solution. When signal amplification was needed, sections were washed and incubated for 1 hour at room temperature in streptavidin–FITC (Jackson ImmunoResearch; 1:500) and counter-stained with DAPI (1 μg/ml). For BrdU detection, sections were treated with 2 M HCl at 37 °C for 15 minutes prior to primary antibody incubation. HCl-treated sections where then equilibrated in borate buffer (0.1 M, pH 8.5) for 10 minutes. For active caspase-3, Tbr1, Tbr2 and TDP-43 detection, antigens were recovered at 80 °C for 30 minutes in Sodium Citrate solution (10 mM, pH 6.0) with 0.05% Tween. Stained sections were embedded in mounting medium containing diazabicyclo-octane (DABCO; Sigma) as an anti-fading agent. Antibodies, dilutions and conditions used for immunolabeling are described in the supplementary tables. Sections were analyzed with an Axioscope (Zeiss) or confocal (Zeiss LSM510) fluorescence microscope. Images were acquired using Axiovision or Zeiss LSM 4.2 (Zeiss) and processed with ImageJ 1.33 or Photoshop CS (Adobe) software.

### iPS cell culture and fluorescent assisted cell sorting

Human iPS cells (Nas2 cells)^48^ were differentiated into cerebral cortex neurons for 39 days according to the protocol from Shi *et al*.^37^. Patient-derived *TDP-43*^*G298S*^ mutant iPS have been characterized and described previously by Alami *et al*.^38^. iPS cells were washed, filtered through a 70 µm cell strainer (Miltenyi Biotec) and sorted on a BD FACS Aria III. Live cells were discriminated by forward and side-scatter (for live cells – from the control) and gated for GFP^-^ (non transfected) or single GFP^+^ populations.

### Quantification and statistical analysis of the data

Randomly selected, stained cells were analyzed with fixed photomultiplier settings on a Zeiss LSM510 confocal microscope (Zeiss). n numbers represent the number of animals used, and images from at least 3 sections per animal were quantified. Data are presented as average percentages of co-labeled cells and statistical comparisons were conducted by two-tailed unpaired Student’s tTest. Significance was established at P < 0.05. In all graphs, error bars are standard deviation (SD).

## Electronic supplementary material

Supplementary Figures and legends
