# Supplementary material for: TDP-43 induces p53-mediated cell death of cortical progenitors and immature neurons
Source: Sci Rep. 2018 May 25;8:8097. doi: 10.1038/s41598-018-26397-2 (PMC5970242; doi:10.1038/s41598-018-26397-2)
Supplement: Supplementary file 1 — Supplementary Figures and legends [file 41598_2018_26397_MOESM1_ESM.docx]

TDP-43 induces p53-mediated cell death of cortical progenitors and immature neurons

Miriam A. Vogt^1¶^, Zahra Ehsaei^1^, Philip Knuckles^2^, Adrian Higginbottom^3^, Michaela S. Helmbrecht^4^, Tilo Kunath^5^, Kevin Eggan^6^, Luis A. Williams^6^, Pamela J. Shaw^3^, Wolfgang Wurst^4^, Thomas Floss^4^, Andrea B. Huber^4§^ and Verdon Taylor^1^

*^1^ Department of Biomedicine, University of Basel, Mattenstrasse 28, 4058 Basel, Switzerland*

*^2^ Friedrich Miescher Institute for Biomedical Research, Maulbeerstrasse 66, 4058 Basel, Switzerland*

*^3^ Sheffield Institute for Translational Neuroscience (SITraN), University of Sheffield, 385A Glossop Road, Sheffield S10 2HQ, UK*

*^4^ Helmholtz Zentrum München, Ingolstädter Landstrasse 1, 85764 Neuherberg, Germany*

*^5^ MRC Centre for Regenerative Medicine, The University of Edinburgh, 5 Little France Drive, Edinburgh, EH16 4UU, UK*

*^6^ Harvard Stem Cell Institute, Harvard University, Howard Hughes Medical Institute, 7 Divinity Avenue, Cambridge, MA 02138, USA*

*^¶^ Current address: Ludwig-Maximilians University Munich, Feodor-Lynen-Strasse 17, 81377 München, Germany*

*^§^ Current address: ETH Zurich, Department of Biosystems Science and Engineering, Mattenstrasse 26, 4058 Basel, Switzerland*

Correspondence should be addressed to V.T. ([verdon.taylor@unibas.ch](mailto:verdon.taylor@unibas.ch)).

# Supplementary Figures and Legends

##
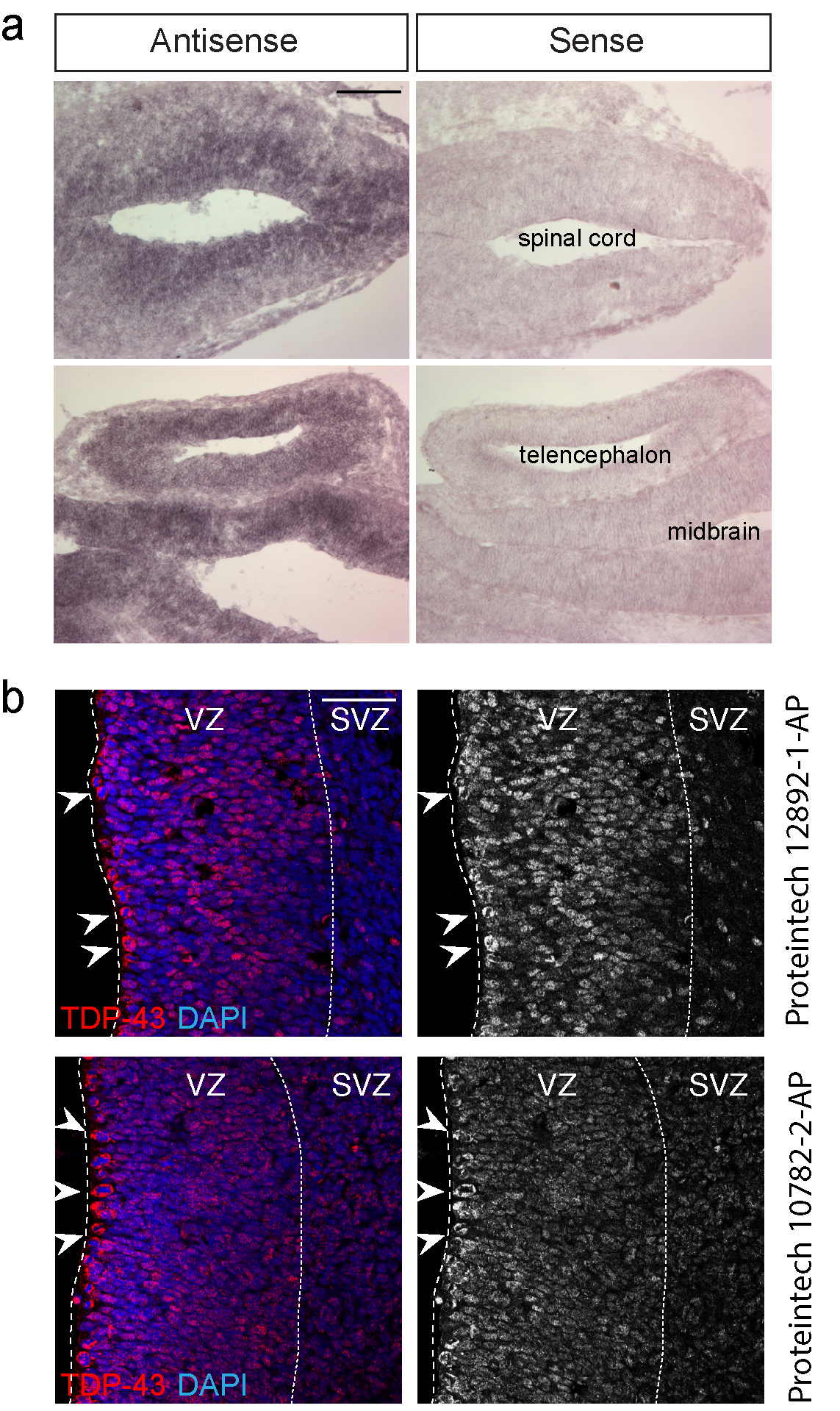


## Supplementary Figure 1: TDP-43 is expressed in neural progenitor cells in the developing CNS

**a.** *In situ* hybridization showing *Tardbp* mRNA expression. *Tardbp* is expressed by neural progenitors of the spinal cord, telencephalon and midbrain of embryonic day (e) 11.5 embryos.

**b.** TDP-43 protein is expressed by ventricular zone (VZ) progenitors in the telencephalon at e14.5 and by cells in mitosis (arrowheads). Immunostaining was performed using two different anti-TDP-43 antibodies from Proteintech: 12892-1-AP and 10782-2-AP. Both antibodies showed the same staining pattern.

Scale bars in a = 100 µm, in b = 25 µm. Dashed line marks the telencephalic vesicle lining. Dotted line marks the border to the subventricular zone (SVZ).


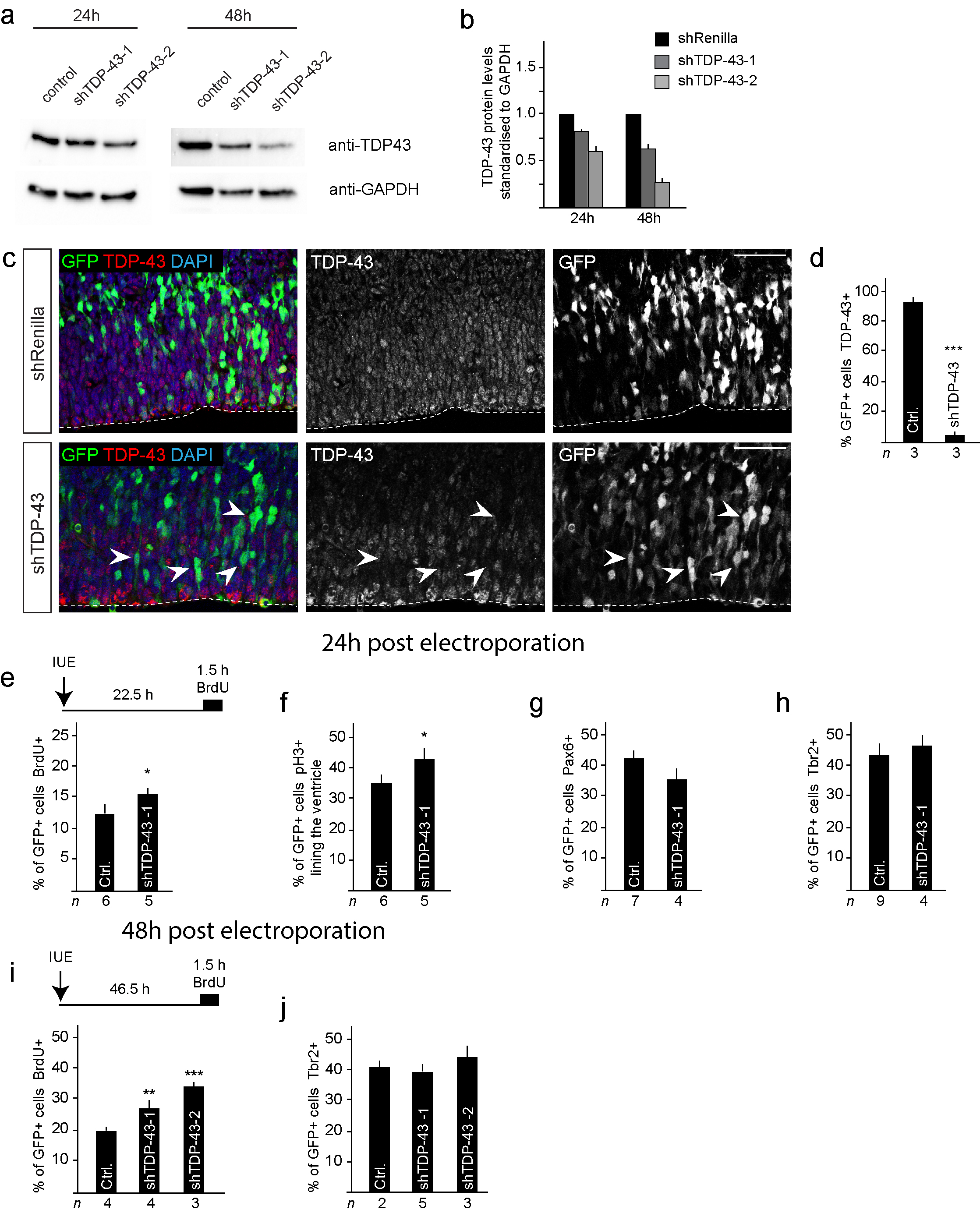


**Supplementary Figure 2: TDP-43 knockdown (KD) results in reduced TDP-43 protein levels**

**a.** TDP-43 KD (shTDP-43) using two different hairpins (shTDP-43-1 and shTDP-43-2) in N2A cells leads to decreased TDP-43 protein levels after 24 and 48 hours. The images are cropped and the full-length blots are shown in the supplementary information.

**b.** Quantification of TDP-43 protein levels after TDP-43 KD relative to GAPDH.

**c, d.** TDP-43 KD (shTDP-43) *in vivo* by *in utero* electroporation (IUE) of neural stem/progenitors at e13.5 and analyzed at e14.5 results in reduced numbers of TDP-43^+^ cells (arrowheads) compared to control (shRenilla) shRNA expression.

**e, i.** Scheme of the BrdU labeling procedure in e13.5 mice with a BrdU pulse 22.5 or 46.5 hours post IUE and 1.5 hours prior to killing. BrdU labeling of TDP-43 KD cells (shTDP-43) is significantly increased. This increased BrdU labeling is more pronounced at 48 hours compared to 24 hours post IUE.

**f.** TDP-43 KD (shTDP-43) results in increased numbers of pH3^+^ mitotic cells lining the ventricle compared to control (shRenilla) shRNA expression 24 hours post IUE.

**g.** Pax6^+^ progenitor cells are reduced by TDP-43 KD (shTDP-43) compared to control (shRenilla) shRNA expression after 24 hours.

**h, j.** TDP-43 KD cells survive in the ventricular zone (VZ) and differentiate to generate Tbr2^+^ basal progenitors in the subventricular zone (SVZ) 24 and 48 hours post IUE.

Scale bar = 25 µm. Dashed line marks the telencephalic vesicle lining. tTest *P<0.05, **<0.01, ***<0.001.


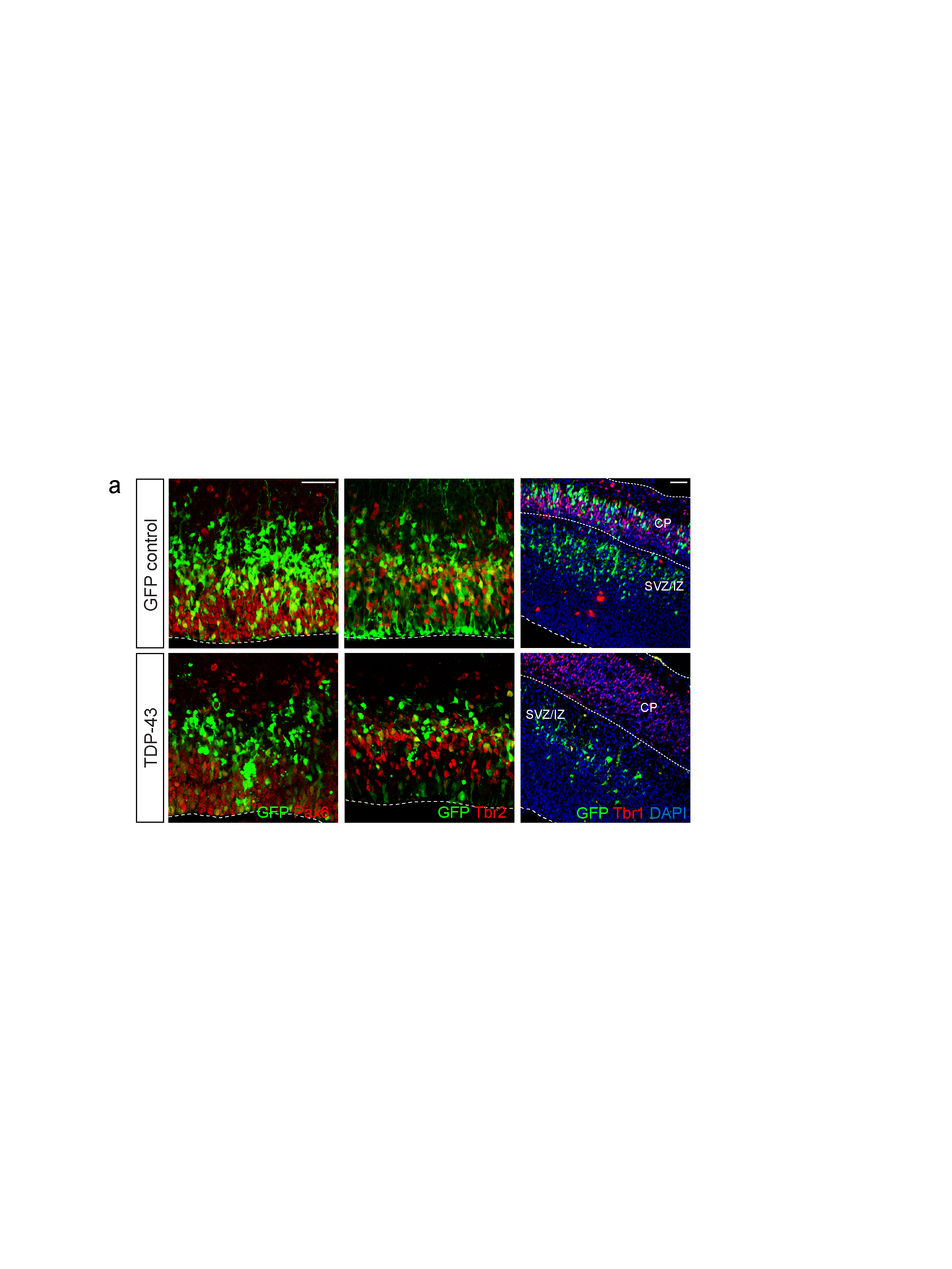


## Supplementary Figure 3: Expression of TDP-43 is detrimental for neural progenitors and decreases neurons in the cortical plate

**a.** TDP-43 overexpression *in vivo* reduces the number of Pax6^+^ progenitors and Tbr2^+^ basal progenitors within 24 hours. TDP-43 overexpressing cells show disrupted non-radial morphology and cellular fragmentation. Unlike GFP expressing control transfected neural progenitors, TDP-43 expressing cells fail to generate Tbr1^+^ cortical neurons and do not migrate to the cortical plate 48 hours post-transfection by *in utero* electroporation.

Scale bar = 25 µm. Dashed line marks the telencephalic vesicle lining. Dotted line marks the border to the CP. Ventricular zone (VZ), subventricular zone (SVZ), intermediate zone (IZ), cortical plate (CP).

##
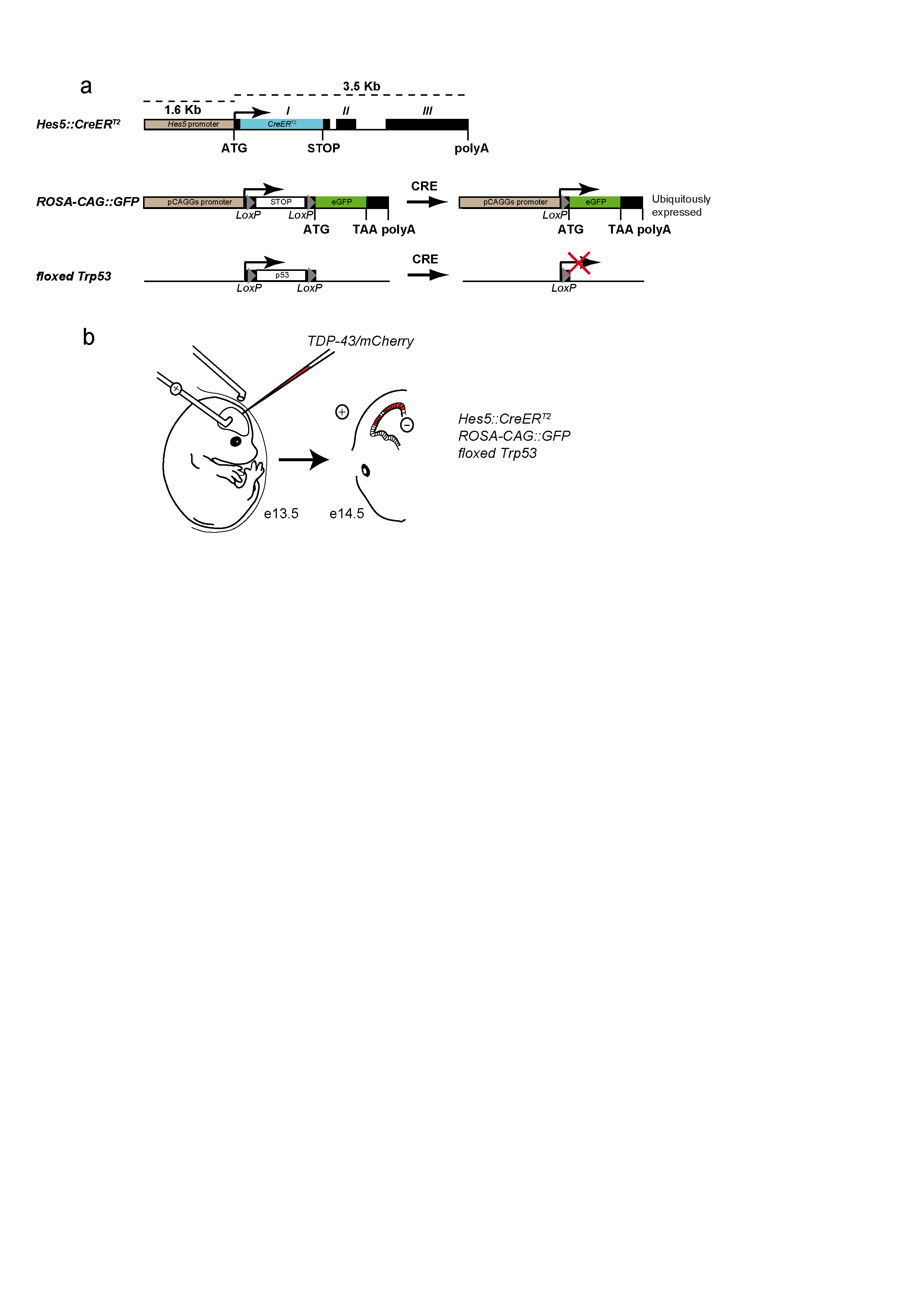


## Supplementary Figure 4: Mosaic deletion of p53 from neural stem/progenitor cells followed by TDP-43 expression

**a.** Scheme of the transgenes used in the conditional *Trp53* gene ablation. Deletion of the floxed *Trp53* loci in neural progenitors was mediated by *Hes5::CreER^T2^* and Tamoxifen induction of pregnant females at 11 days post-coitum. Recombination of the *ROSA-CAG::GFP* allele results in ubiquitous expression of GFP in Cre-expressing cells and their progeny enabling lineage-tracing.

**b.** Scheme of the *in utero* electroporation experimental procedure. Mothers were induced with Tamoxifen at 11 days post-coitum. *Hes5::CreER^T2^*, *ROSA-CAG::GFP,* *p53::floxed* embryos were *in utero* electroporated with *TDP-43* expression vectors and *pCAGGs::mCherry* (vectors as transfection reporter) at e13.5. Embryos were sacrificed and analyzed one day later at e14.5.


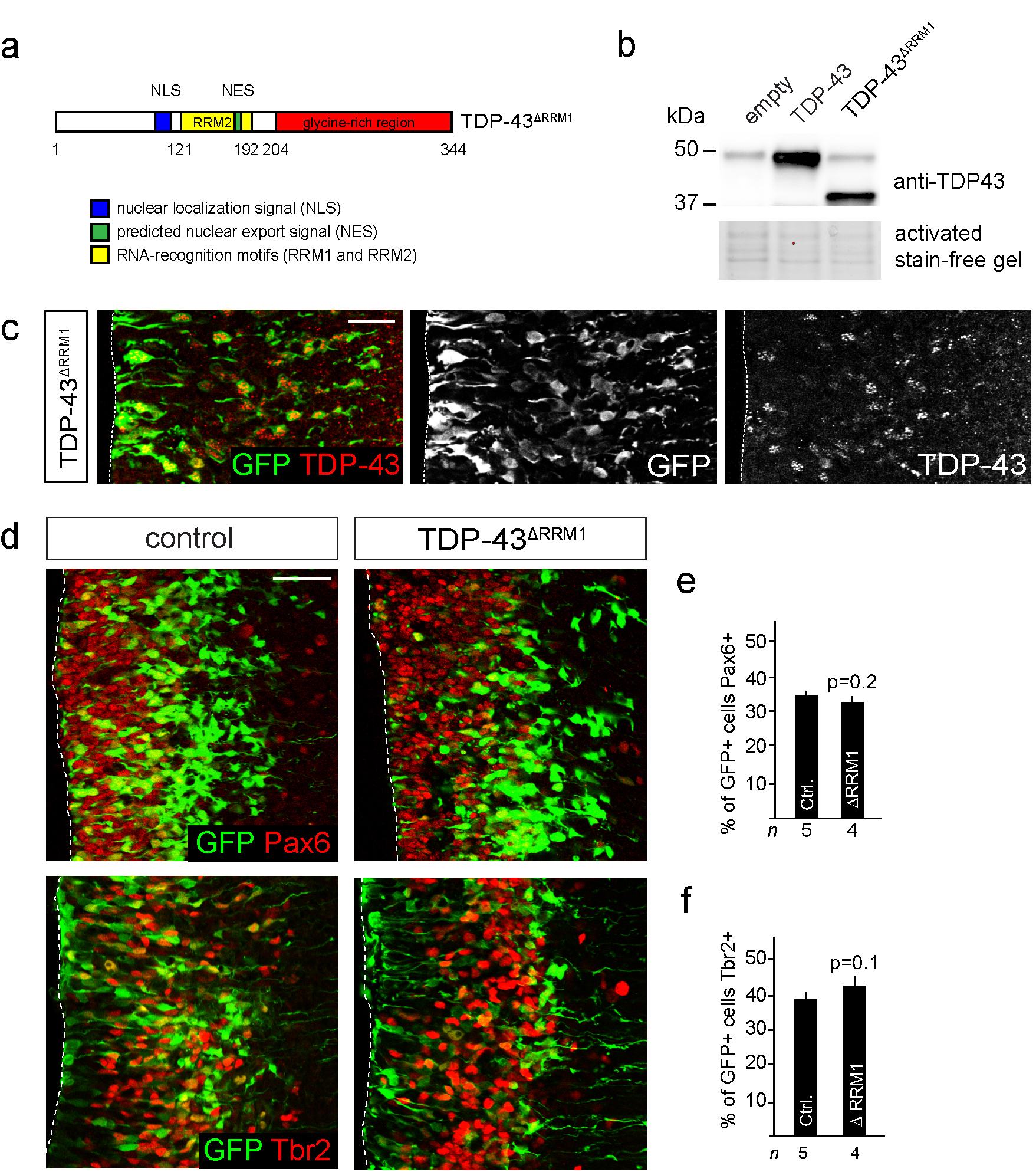


## Supplementary Figure 5: Toxic effect of TDP-43 is dependent on its binding to RNA

**a.** Scheme of the TDP-43^∆RRM1^ protein. The RRM1 domain is critical for RNA binding.

**b.** Transfection of neural progenitors with TDP-43^∆RRM1^ expression vectors results in a 3.7-fold overexpression of TDP-43^∆RRM1^ compared to endogenous TDP-43 levels. The images are cropped and the full-length blots are shown in the supplementary information.

**c.** Expression of TDP-43^∆RRM1^ does not result in apoptosis. TDP-43^∆RRM1^ expressing cells show a normal radial morphology and migrate to the subventricular zone. Transfected TDP-43^∆RRM1^ expressing cells show formation of TDP-43 aggregates in the nucleus and cytoplasm.

**d.** TDP-43^∆RRM1^ does not affect Pax6 and Tbr2 expression by progenitors *in vivo* and does not result in signs of cell death.

**e.** The number of Pax6^+^ cells is not changed by the expression of TDP-43^∆RRM1^. tTest P=0.2.

**f.** The number of Tbr2^+^ cells is not changed by the expression of TDP-43^∆RRM1^. tTest P=0.1.

Scale bars = 25 µm. Dashed line marks the ventricular lining.


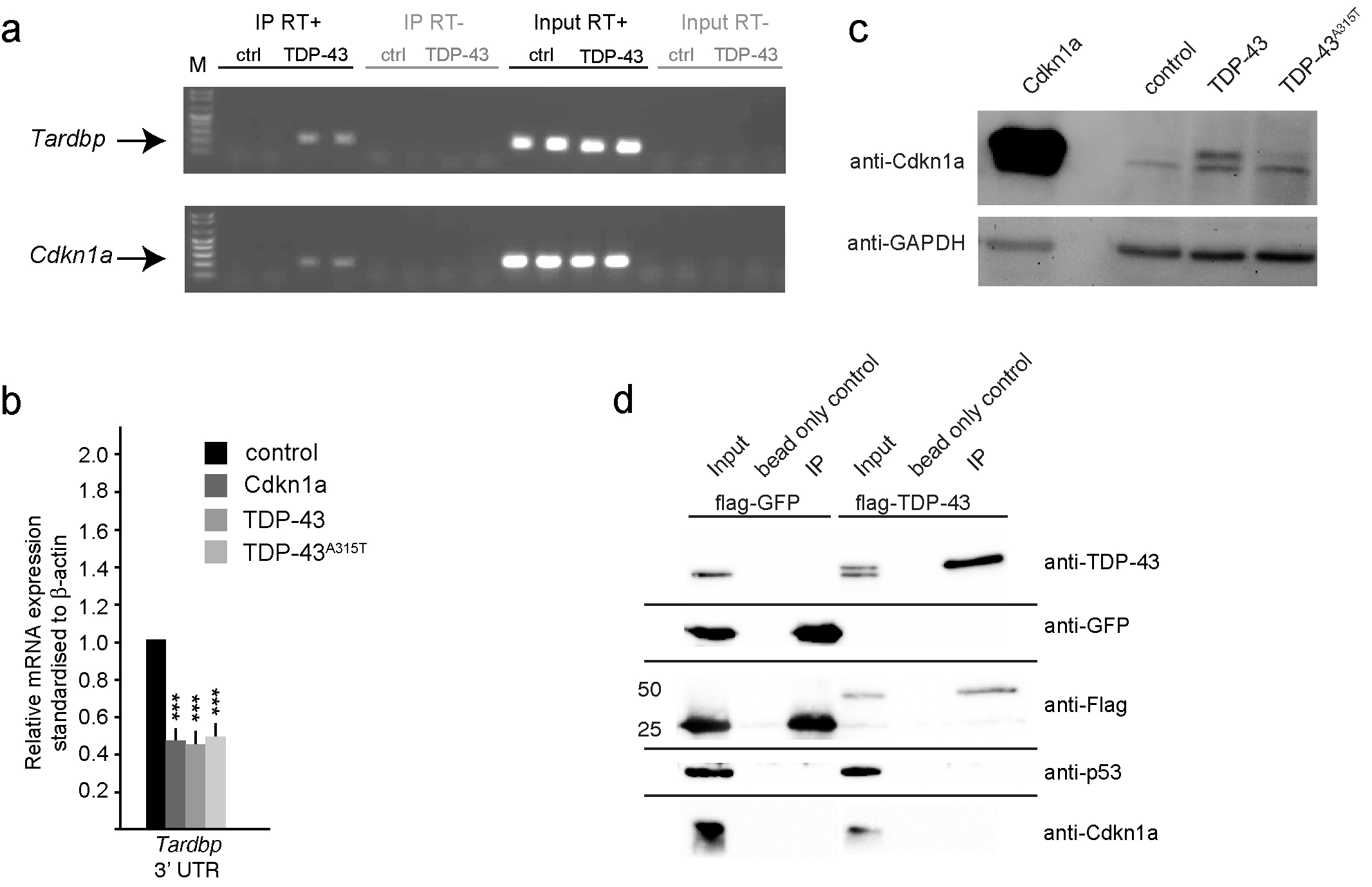


## Supplementary Figure 6: TDP-43 associates with *Cdkn1a* mRNA and regulates Cdkn1a expression

**a.** Agarose gel analysis of the amplicons for *Tardbp* and *Cdkn1a* mRNA after CLIP with Flag-tagged TDP-43 and RT-PCR. Flag-tagged GFP CLIP pull-down was used as a control. The agarose gels are representative of experiments performed in duplicate (2 lanes per CLIP). CLIP reverse transcription^+^ (RT^+^) shows amplification of precipitated *Tardbp* and *Cdkn1a* RNAs. CLIP RT^-^ and Input RT^+^ are controls for RNA amplification, absence of genomic DNA contamination and amounts of input RNA in the CLIP experiments for each sample. The specificity of the amplicons was verified by sequencing.

**b.** qRT-PCR analysis of N2A cells transfected with GFP (control), TDP-43 or TDP-43^A315T^. Note the decrease in endogenous *Tardbp* 3’UTR after TDP-43 and TDP-43^A315T^ expression.

**c.** TDP-43 and TDP-43^A315T^ expression in N2A cells increases total Cdkn1a protein expression. The images are cropped and the full-length blots are shown in the supplementary information.

**d.** Co-immunoprecipitation analysis showing that TDP-43 does not interact with p53 or Cdkn1a proteins in N2A cells. The images are cropped and the full-length blots are shown in the supplementary information. tTest ***P<0.001.


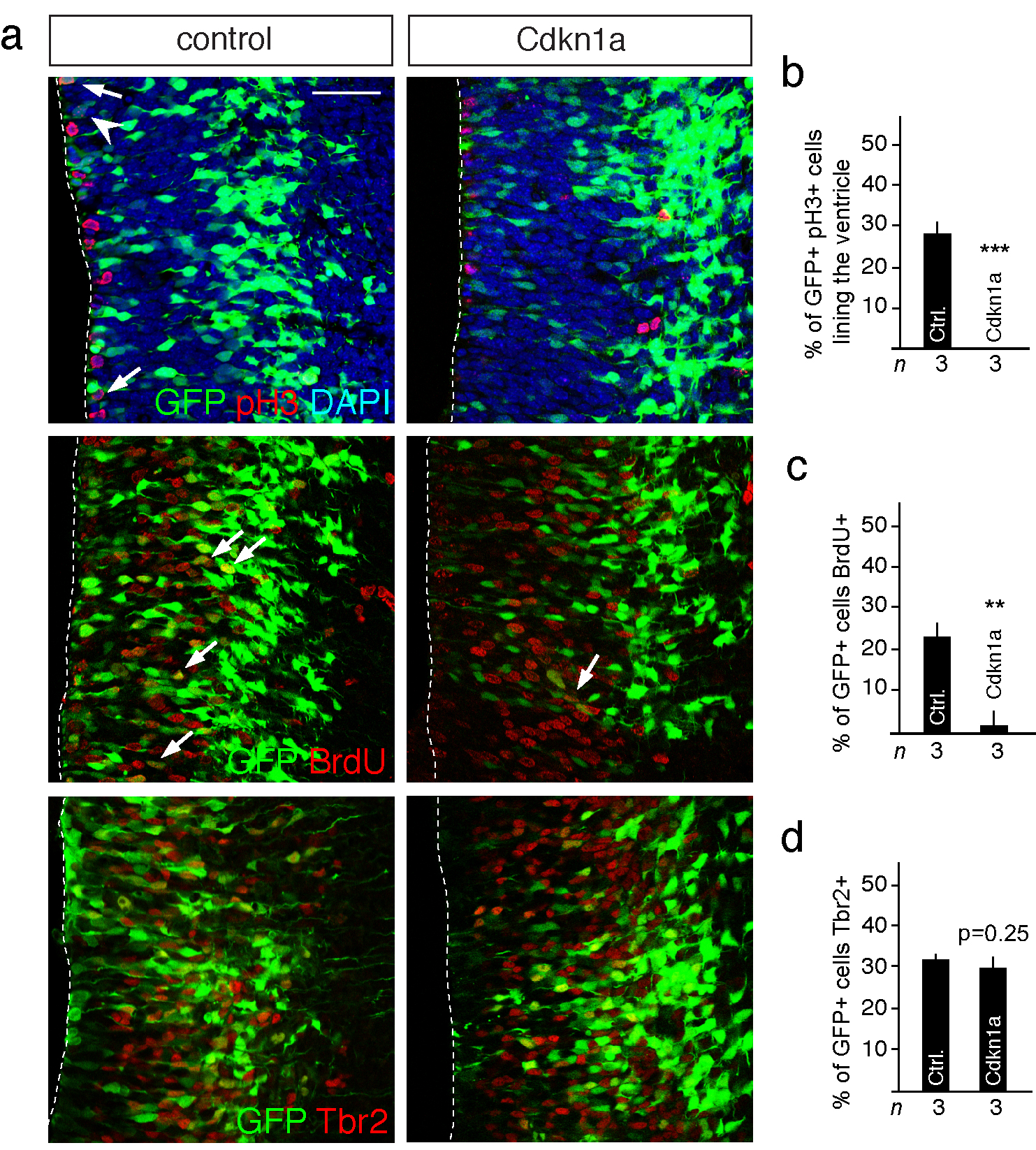


## Supplementary Figure 7: Cdkn1a overexpression *in vivo* does not induce apoptosis

**a.** Expression of Cdkn1a by *in utero* electroporation does not induce cell death of neural progenitors *in vivo*. However, Cdkn1a expressing ventricular zone (VZ) progenitors migrated to the subventricular zone (SVZ). In addition, the number of cells in M-phase (pH3^+^) and in S-phase (BrdU^+^) of the cell cycle is reduced following Cdkn1a overexpression (arrows). Although Cdkn1a induces exit from the VZ, the cells do not up regulate the basal progenitor marker Tbr2.

**b.** Quantification of pH3 expressing cells following expression of Cdkn1a compared to GFP expressing control cells.

**c.** Quantification of BrdU incorporating cells following expression of Cdkn1a compared to GFP expressing control cells.

**d.** Quantification of Tbr2 expressing cells following expression of Cdkn1a compared to GFP expressing control cells. tTest P=0.25

Scale bar = 25 µm. Dashed line marks the ventricular lining. tTest **P<0.01, ***P<0.001.

**
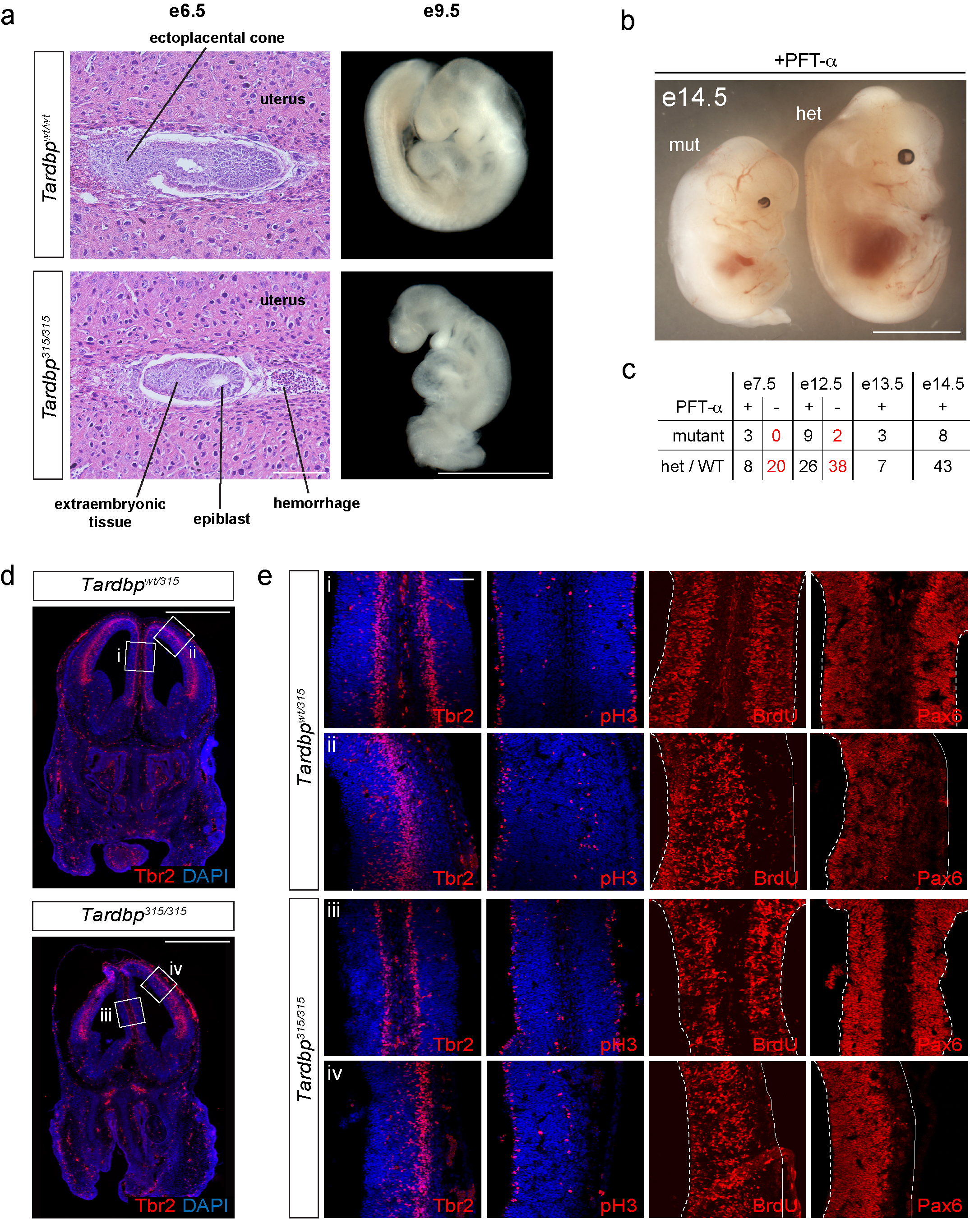
**

**Supplementary Figure 8: TDP-43 and human mutant TDP-43^A315T^ toxicity during embryogenesis is partially rescued by inhibition of p53**

**a.** Most mice homozygous for a knock-in of human *TARDBP^315^* into the endogenous *Tardbp* gene locus (*Tardbp^315/315^*) die *in utero* before e9.5. Embryos surviving to e9.5 are severely developmentally retarded, morphologically abnormal and will be resorbed. At e6.5 post-implantation embryos form an epiblast but have signs of hemorrhage in the extra-embryonic space.

**b.** PFT-α rescued *Tardbp^315/315^* mutants are slightly smaller than their heterozygous (Het) and wild type (WT) litter siblings.

**c.** Most *Tardbp^315/315^* mutants die before e9.5 and are underrepresented in pregnancies compared to *Tardbp^wt/315^* (Het) or *Tardbp^wt/wt^* (WT) litter mates (red numbers e7.5-e12.5). Treatment of timed-mated pregnant females from *Tardbp^wt/315^* intercrosses with PFT-α rescues *Tardbp^315/315^* mutants (black numbers).

**d.** The brains of PFT-α rescued *Tardbp^315/315^* e14.5 embryos are structurally normal and show correct marker expression.

**e.** Expression of Tbr2, pH3, BrdU and Pax6 in the brain of *Tardbp^315/315^* embryos after PFT-α rescue. The images are taken from the regions i-iv shown in d. Cortical layering and proliferation of rescued *Tardbp^315/315^* embryos at e14.5 is indistinguishable from control embryos.

Scale bars = 50 µm at e6.5 and 500 µm at e9.5, 1 mm in b, 500 µm in d, 25 µm in e. Dashed line marks the telencephalic vesicle lining and solid line the outer surface of the telencephalon.

# Supplementary Information full-length blots

#
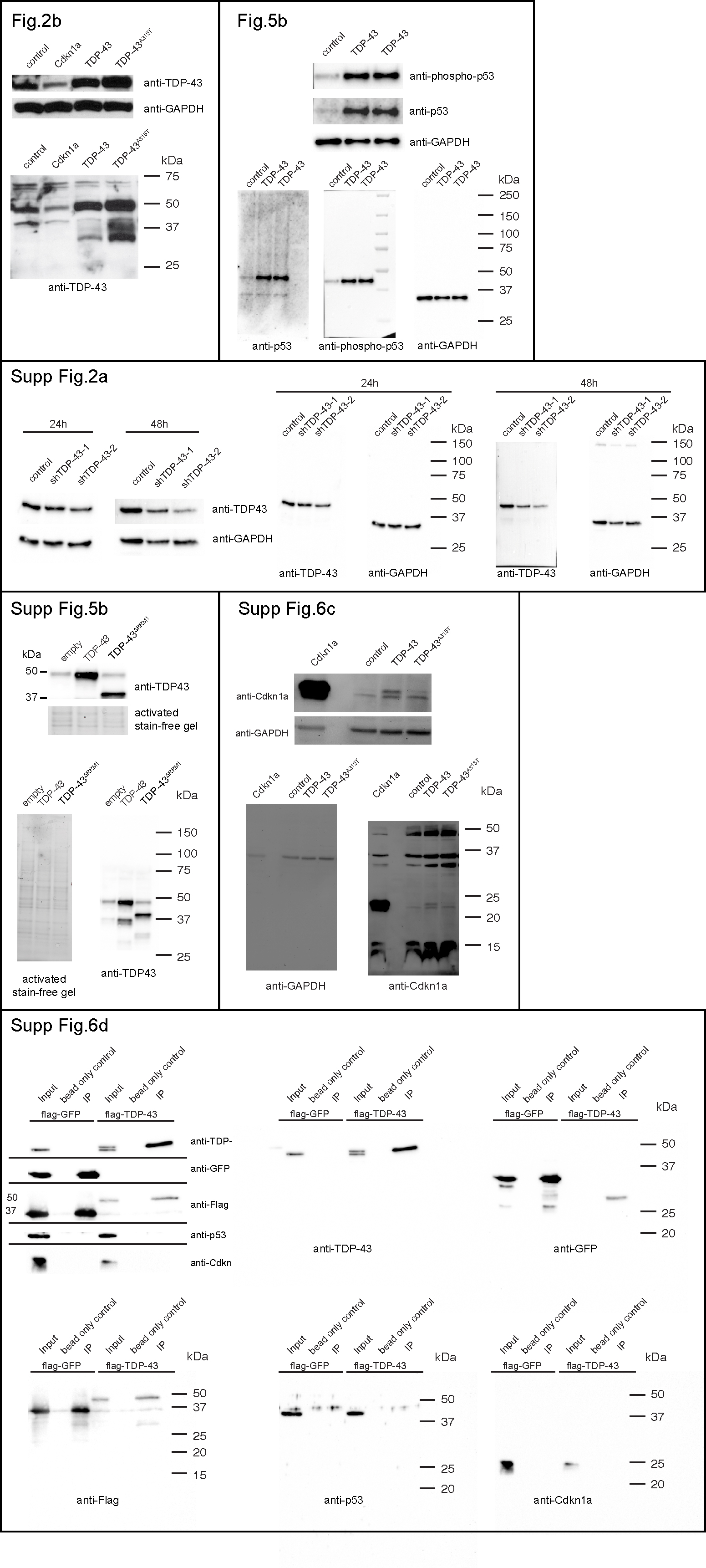


**Images of full-length immunoblots.** The cropped blots from the main Figures and Supplementary Figures are shown together with their corresponding full-length blots.

# Supplementary Tables of Antibodies and Primers

**Primary Antibodies for Immunostaining**

| **Antigen** | **Species** | **Dilution** | **Source** | **Catalog number** |
| --- | --- | --- | --- | --- |
| **Anti-cleaved Caspase3** | Rabbit | 1:200 | Cell signaling | 9664S |
| **Anti-Cdkn1a** | Rabbit | 1:500 | Abcam | ab7960 |
| **Anti-βIII-Tubulin** | Mouse | 1:400 | Sigma | T8660 |
| **Anti-Pax6** | Rabbit | 1:300 | Covance | PRB-278P |
| **Anti-pH3** | Rabbit | 1:300 | Millipore | 06-570 |
| **Anti-Tbr1** | Rabbit | 1:500 | Abcam | AB31940-100 |
| **Anti-Tbr2** | Rabbit | 1:500 | Abcam | AB23345 |
| **Anti-TDP-43** | Rabbit | 1:500 | Proteintech | 12892-1-AP |
| **Anti-TDP-43** | Rabbit | 1:500 | Proteintech | 10782-2-AP |

**Primary Antibodies for Immunoblotting**

| **Antigen** | **Species** | **Dilution** | **Source** | **Catalog number** |
| --- | --- | --- | --- | --- |
| **Anti-Flag** | Mouse | 1:2000 | Sigma | F3165 |
| **Anti-GAPDH** | Mouse | 1:10000 | Calbiochem | CB1001 |
| **Anti-GFP** | Mouse | 1:1000 | Roche | 11814460001 |
| **Anti-Cdkn1a** | Rabbit | 1:100 | Abcam | ab7960 |
| **Anti-Phospho-p53 (Ser15)** | Rabbit | 1:1000 | Cell Signalling | 9284 |
| **Anti-p53** | Rabbit | 1:1000 | Abcam | ab26 |
| **Anti-TDP-43** | Rabbit | 1:2000 | Proteintech | 12892-1-AP |

**Secondary Antibodies for Immunostaining**

| **Antigen** | **Species** | **Dilution** | **Source** | **Catalog number** |
| --- | --- | --- | --- | --- |
| **Alexa488 conjugated anti-mouse Immunoglobulin (affinity purified and adsorbed)** | Donkey | 1:1000 | Jackson ImmunoResearch | 715546151 |
| **Cy3 conjugated anti-mouse Immunoglobulin (affinity purified and adsorbed)** | Donkey | 1:1000 | Jackson ImmunoResearch | 715165151 |
| **Alexa488 conjugated anti-Rabbit Immunoglobulin (affinity purified and adsorbed)** | Donkey | 1:1000 | Jackson ImmunoResearch | 711545152 |
| **Cy3 conjugated anti-rabbit Immunoglobulin (affinity purified and adsorbed)** | Donkey | 1:1000 | Jackson ImmunoResearch | 711165152 |
| **Cy3 conjugated anti-rat Immunoglobulin (affinity purified and adsorbed)** | Donkey | 1:1000 | Jackson ImmunoResearch | 712160153 |
| **Cy5 conjugated anti-rabbit Immunoglobulin (affinity purified and adsorbed)** | Donkey | 1:1000 | Jackson ImmunoResearch | 711605192 |

**Secondary Antibodies for Immunoblotting**

| **Antigen** | **Species** | **Dilution** | **Source** | **Catalog number** |
| --- | --- | --- | --- | --- |
| **Peroxidase-conjugated anti-mouse Immunoglobulin** | Donkey | 1:10000 | Jackson ImmunoResearch | 715035152 |
| **Peroxidase-conjugated anti-rabbit Immunoglobulin** | Donkey | 1:10000 | Jackson ImmunoResearch | 711035152 |

**PCR primers**

| **qPCR primers** | **Forward 5'-3'** | **Reverse 5'-3'** |
| --- | --- | --- |
| **β-actin** | aggtgacagcattgcttctg | gggagaccaaagccttc |
| **Bax** | tgaagacagggcctttttg | aattcgccggagacactcg |
| **Bcl2** | cctgtggtcatggatctgtt | ggaagaccaggctttcttgt |
| **Bbc3** | agcagcacttcgcgtcgcc | cctgggtaaggggaggagt |
| **GAPDH** | tccatgacaactttggcattgtgg | gttgctgttgaagtcgcaggagac |
| **p21-3’UTR** | aaggccagctaggatgacag | agagacccacaggagaggtg |
| **p53-3’UTR** | cccagcgaaattctatccag | cagacaggctttgcagaatg |
| **TDP-43-3’UTR** | gcccacaaactgaggggataa | tgtcctcctgcacacaagtc |
